# Down regulation of macrophage IFNGR1 exacerbates systemic *L*. *monocytogenes* infection

**DOI:** 10.1371/journal.ppat.1006388

**Published:** 2017-05-22

**Authors:** Emily M. Eshleman, Christine Delgado, Staci J. Kearney, Rachel S. Friedman, Laurel L. Lenz

**Affiliations:** 1 Department of Immunology and Microbiology, University of Colorado School of Medicine, Aurora, CO United States of America; 2 Department of Biomedical Sciences, National Jewish Health, Denver, CO United States of America; DUMC, UNITED STATES

## Abstract

Interferons (IFNs) target macrophages to regulate inflammation and resistance to microbial infections. The type II IFN (IFNγ) acts on a cell surface receptor (IFNGR) to promote gene expression that enhance macrophage inflammatory and anti-microbial activity. Type I IFNs can dampen macrophage responsiveness to IFNγ and are associated with increased susceptibility to numerous bacterial infections. The precise mechanisms responsible for these effects remain unclear. Type I IFNs silence macrophage *ifngr1* transcription and thus reduce cell surface expression of IFNGR1. To test how these events might impact macrophage activation and host resistance during bacterial infection, we developed transgenic mice that express a functional FLAG-tagged IFNGR1 (fGR1) driven by a macrophage-specific promoter. Macrophages from fGR1 mice expressed physiologic levels of cell surface IFNGR1 at steady state and responded equivalently to WT C57Bl/6 macrophages when treated with IFNγ alone. However, fGR1 macrophages retained cell surface IFNGR1 and showed enhanced responsiveness to IFNγ in the presence of type I IFNs. When fGR1 mice were infected with the bacterium *Listeria monocytogenes* their resistance was significantly increased, despite normal type I and II IFN production. Enhanced resistance was dependent on IFNγ and associated with increased macrophage activation and antimicrobial function. These results argue that down regulation of myeloid cell IFNGR1 is an important mechanism by which type I IFNs suppress inflammatory and anti-bacterial functions of macrophages.

## Author summary

Interferon (IFN)γ promotes host resistance to invasive bacterial infections, but type I IFNs suppress macrophage activation and increase susceptibility to *L*. *monocytogenes* and other pathogenic bacteria. IFNγ promotes resistance by potently activating macrophages to become pro-inflammatory and antimicrobial. Type I IFNs have the opposite effect on resistance during many bacterial infections. They can also suppress macrophage responsiveness to IFNγ through the down regulation of its signaling receptor (IFNGR). Here, we developed an experimental system to investigate the significance of IFNGR1 down regulation as a mechanism contributing to pro-bacterial effects of type I IFNs. A transgenic mouse (fGR1) was generated in which macrophages resist type I IFN-induced down regulation of IFNGR1. We report that macrophages from fGR1 mice retain responsiveness to IFNγ when exposed to type I IFNs. fGR1 mice thus have increase resistance to systemic infection by *L*. *monocytogenes*. This resistance is dependent on IFNγ and associates with increased evidence of macrophage activation and anti-bacterial activity. Our findings thus suggest modulation of IFNGR1 is an important mechanism by which type I IFNs increase host susceptibility to bacterial infections.

## Introduction

Interferons (IFN) are critical mediators of host immunity. These cytokines are classified into two major groups: type I and type II. Type I IFNs are a family of homologous proteins including various IFNα subtypes and a single IFNβ [1,2]. They are synthesized and secreted in response to ligation of transmembrane or cytosolic pattern recognition receptors (PRR) by a variety of microbial products [3]. All type I IFNs signal through a commonly expressed heterodimeric receptor, IFNAR. Ligation of IFNAR activates TYK2 and JAK1 kinases to phosphorylate signal transducers and activator of transcription (STAT) proteins 1 and 2 [2,4]. These activated STATs associate with IRF9 to induce transcription of numerous IFN-stimulated genes (ISGs) whose products restrict viral replication [1]. Type I IFNs additionally impact cell survival and can have anti-inflammatory effects [3]. They are used therapeutically in treatment of chronic viral infections, cancers, and the autoimmune disease multiple sclerosis.

IFNγ is the only type II IFN. It signals through its own ubiquitously-expressed heterodimeric receptor (IFNGR) comprised of IFNGR1 and IFNGR2 subunits. IFNγ binds directly to IFNGR1 to aggregate receptor subunits such that one signaling complex contains two IFNGR1 and two IFNGR2 chains [2,4,5]. This aggregation activates JAK1 and JAK2 kinases to phosphorylate STAT1 proteins [2,4]. Phosphorylated STAT1 proteins homodimerize and translocate to the nucleus to initiate transcription of IFNγ-activated genes (GAG) [2,4]. Several antiviral proteins are both ISGs and GAGs, but IFNγ uniquely stimulates macrophages to express certain GAGs that promote inflammatory responses (e.g. TNF-α, IL-12, CXCL9, CXCL10), contribute to antigen processing and presentation to T cells (e.g. MHC class I and II, CD40, CD80, CD86, the high-affinity Fc receptor CD64), and promote macrophage bactericidal activity (e.g. nitric oxide synthase 2, NOS2, and NADPH oxidase subunits) [6,7]. By inducing expression of these and other GAGs, IFNγ drives M1-type pro-inflammatory and microbicidal activation in macrophages.

IFNγ is vital for host resistance to bacterial infections, while type I IFNs have the opposite effect of increasing host susceptibility. Thus, mice lacking IFNγ, IFNGR1, or critical signaling components such as STAT1 are exquisitely susceptible to infections by *Mycobacteria tuberculous*, *Listeria monocytogenes* and other pathogens [8–12]. By contrast, mice unresponsive to type I IFNs are 100–1000 times more resistant to mucosal and systemic infections by *M*. *tuberculosis*, *M*. *leprae*, *L*. *monocytogenes*, and numerous other pathogenic bacteria [3,4,13,14]. These data indicate type I IFNs normally increase susceptibility. Precisely how they do so is debated. It is clear type I IFNs reduce production of several myeloid cell-active cytokines and chemokines with presumed protective functions and increase expression of anti-inflammatory factors [15–20]. Thus, detrimental effects of type I IFNs appear to impair myeloid cell immunity [3,4,13,14]. However, it is not agreed how type I IFNs mediate this suppression of myeloid cells. Type I IFNs may primarily act through induction of IL-10 [15–18,20–22], or by suppressing inflammasome activation and IL-1 production in myeloid cells responding to infection [23–25]. Alternatively, we previously suggested type I IFNs may suppress macrophage activation due to their ability to silence myeloid cell *ifngr1* transcription and down regulate macrophage IFNGR1 [26–28].

To distinguish between the above models and test the importance of type I IFN driven down regulation of IFNGR1, we developed a transgenic mouse line in which a functional FLAG-tagged IFNGR1 (fGR1) is selectively expressed in macrophages using the well-characterized *c-fms* promoter [29,30]. Staining for the FLAG epitope revealed the fGR1 transgenic IFNGR1 was expressed at very low levels selectively on macrophages and inflammatory monocytes. Total cell surface IFNGR1 on macrophages was equivalent for unstimulated control and fGR1 mice. Control and fGR1 macrophages also responded similarly to stimulation with IFNγ. When stimulated with type I IFN, fGR1 macrophages showed similar induction of canonical responses as control cells but selectively retained more cell surface IFNGR1. The ability to resist IFNGR1 down regulation enabled cultured fGR1 macrophages to respond better to IFNγ following stimulation with type I IFN. In fGR1 mice, macrophages also showed increased evidence of M1-type activation during systemic infection with the type I IFN-stimulating bacterial pathogen *L*. *monocytogenes*. Further, bacterial burdens were significantly reduced in the fGR1 mice. Heightened resistance of fGR1 mice was dependent on host production of IFNγ and associated with significantly increased bacterial uptake and containment in lysosomes, as measured by reduced overall bacterial burdens in fGR1 cells and increased co-localization of GFP expressing *L*. *monocytogenes* with the lysosomal marker LAMP-1. These data indicate that preventing type I IFN stimulated down regulation of myeloid cell IFNGR1 during bacterial infection increases host resistance by permitting increased macrophage responsiveness to IFNγ and thus increased macrophage activation and bactericidal functions. We conclude that down regulation of myeloid cell IFNGR1 contributes significantly to the enhanced susceptibility associated with type I IFN production during bacterial infection.

## Results

### fGR1 transgene is restricted to macrophages and mediates responsiveness to IFNγ

A *c-fms* promoter construct driving expression of cDNA encoding IFNGR1 with an N-terminal FLAG-epitope tag was used to generate fGR1 transgenic mice. The endogenous *c-fms* promoter drives expression of the CSF1R, which is selectively expressed in myeloid cells [30]. Transgenic mice developed normally and transmitted the fGR1 transgene with a Mendelian inheritance pattern. Leukocyte frequencies were similar in the spleen, peritoneum, liver, lungs, and intestines of naïve WT C57Bl/6 and fGR1 mice, as was expression of activation markers such as MHC II (S1A and S1B Table). Thus, fGR1 expression failed to notably impact hematopoietic cell development or activation in the absence of infection.

**Fig 1:**
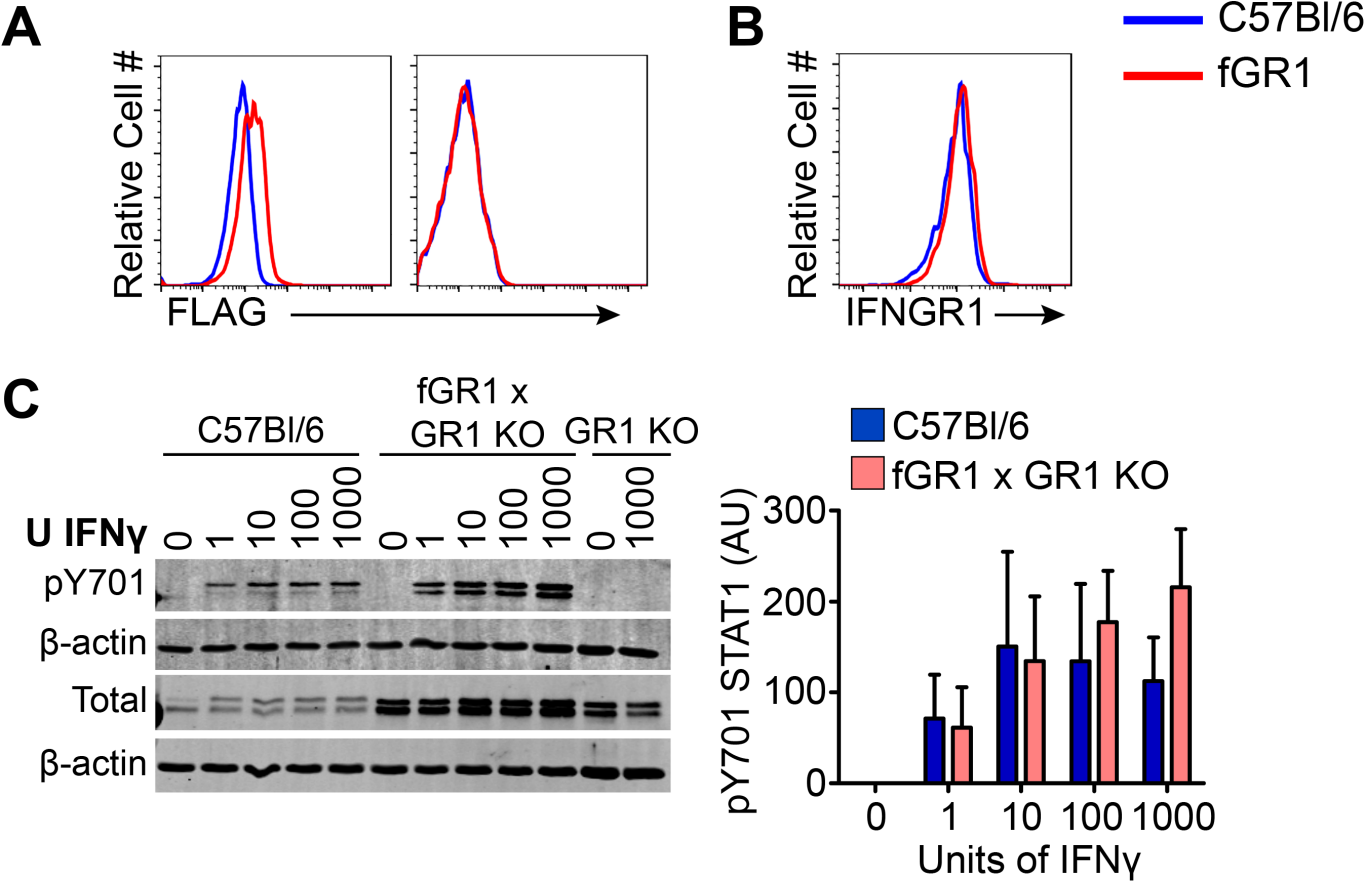
fGR1 expression is low and restricted to macrophages. Naïve peritoneal cells were isolated from WT C57Bl/6 (Blue) and fGR1+ (Red) mice. A) FLAG expression was analyzed on both macrophage (CD90.2 -, CD11b^hi^, F480^hi^) (Left) and T cell (CD90.2+) (Right) populations by flow cytometry. B) Surface expression of IFNGR1 on unstimulated naïve peritoneal macrophages from both mouse genotypes. C) Naïve peritoneal macrophages from C57Bl/6, fGR1 x GR1 KO, or B6.*ifngr1*^*-/-*^ (GR1 KO) mice were stimulated with increasing concentrations of IFNγ for 30 min. Phosphorylation of Y701 STAT1 was detected by immunoblot. Graph depicts density of pSTAT1 bands normalized to the loading control β-actin, Arbitrary Units (AU). (n = 3 independent experiments).

Commercial and custom antibodies directed at the FLAG epitope stained macrophages and monocytes but not other cell populations from the fGR1 mice. Fig 1A illustrates typical anti-FLAG staining on gated CD90.2-, CD11b^hi^, F480^hi^ macrophages (Left) and CD90.2^+^ (Thy1^+^) T cells (Right) isolated from the peritoneum of naïve WT and fGR1 mice. Staining for fGR1 was also weak and restricted to monocytes and macrophages in all other examined tissues (S1 Fig). Total cell surface IFNGR1 staining was equivalent on fGR1 and WT C57Bl/6 macrophages (Fig 1B), indicating fGR1 did not increase overall cell surface IFNGR1. To confirm responsiveness of the fGR1 transgenic receptor to IFNγ, the fGR1 transgene was crossed to B6.*ifngr1*^-/-^ mice. Macrophages from the resulting fGR1 x *ifngr1*^-/-^ (fGR1 x GR1 KO) mice were then stimulated with increasing concentrations of IFNγ. Phosphorylation of STAT1 (tyrosine 701) was equivalent in fGR1 x GR1 KO and WT C57Bl/6 macrophages, even though total STAT1 was observed to be increased in *ifngr1*^*-/-*^ macrophages regardless of whether they also expressed fGR1 (Fig 1C). Collectively, these data demonstrated myeloid cell-restricted expression and functionality of the FLAG-tagged IFNGR1 in the fGR1 mice.

### fGR1 expression does not alter basal IFN signaling

**Fig 2:**
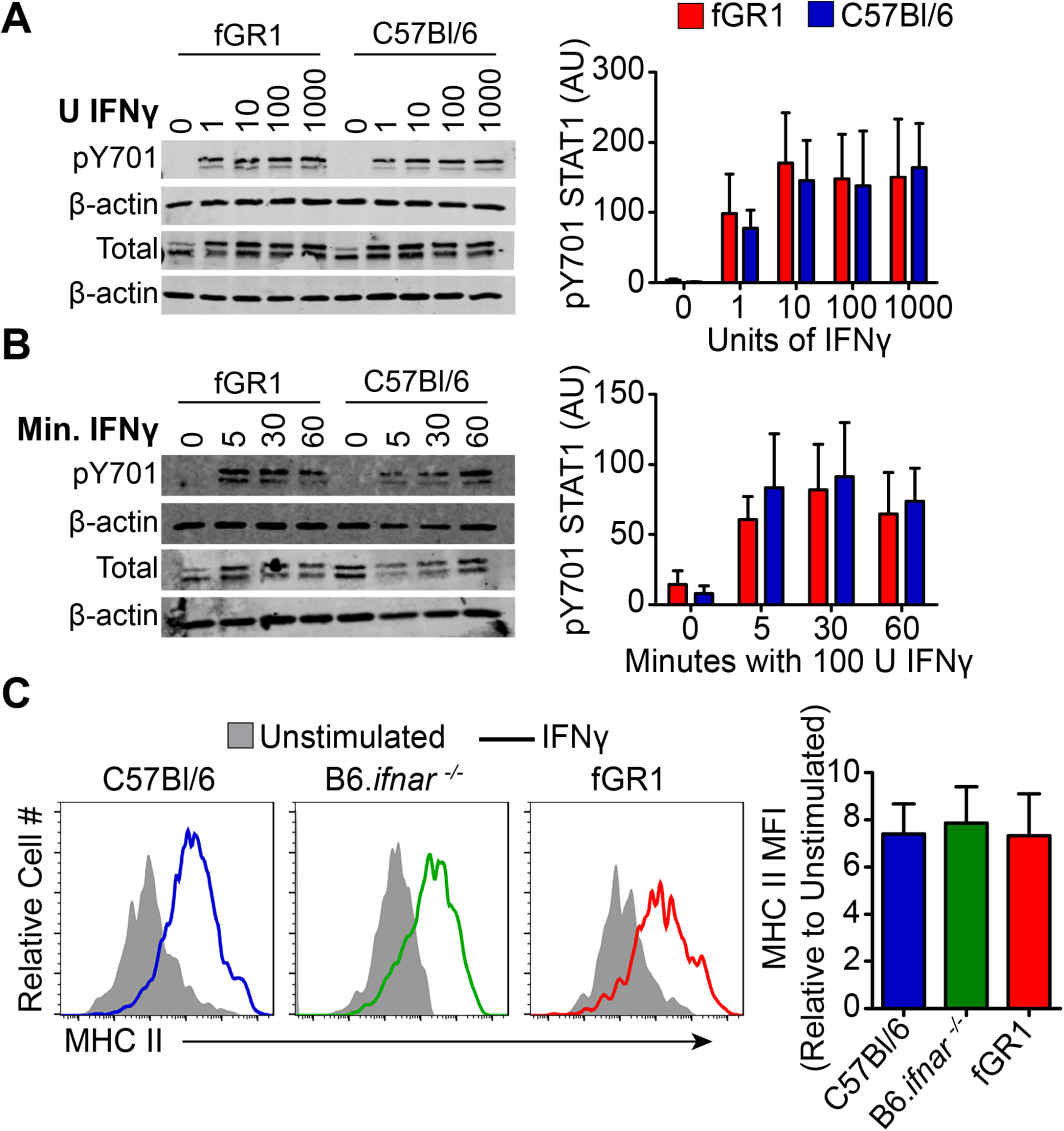
fGR1 expression does not alter basal IFNγ responsiveness. Peritoneal macrophages from naïve fGR1 and WT mice were A) stimulated for 30 min with increasing concentrations of IFNγ, B) 100 U/mL of IFNγ for 5, 30, 60 min and pY701 STAT1 determined by western blots. pSTAT1 blots were normalized to β-actin, Arbitrary Units (AU). C) Naïve peritoneal macrophages (CD90.2 -, CD11b^hi^, F480^hi^) from IFNAR KO (green), C57Bl/6 (blue), and fGR1 (red) were stimulated with 100 U/mL of IFNγ for 24 hrs and MHC II up regulation evaluated by flow cytometry. MFIs were normalized to their respective unstimulated controls and pooled from multiple experiments (n = 3 independent experiments, 1–3 mice per experiment).

We next evaluated STAT1 Y701 phosphorylation early after IFNγ stimulation of WT and fGR1 peritoneal macrophages. WT and fGR1 macrophages responded similarly to 30 min stimulation with various concentrations of IFNγ (Fig 2A). Likewise, kinetics of STAT1 phosphorylation were not affected by fGR1 expression, as determined by quantitative immunoblotting of lysates from WT and fGR1 macrophages 5, 30, or 60 min after treatment with 100 units (U)/mL of IFNγ (Fig 2B). As an indicator of later responses to IFNγ, we measured induction of MHC II in macrophages. MHC II is a GAG whose induction requires multiple rounds of ligand-receptor binding and subsequent receptor internalization [31]. Overnight stimulation with IFNγ induced similar upregulation of cell surface MHC II in WT, B6.*ifnar1*^-/-^, and fGR1 macrophages (Fig 2C). Thus, in the absence of type I IFN stimulation fGR1 expression did not appreciably alter early or delayed macrophage responsiveness to IFNγ.

### fGR1 expression blunts loss of IFNGR1 expression and preserves sensitivity to IFNγ in macrophages treated with type I IFNs

**Fig 3:**
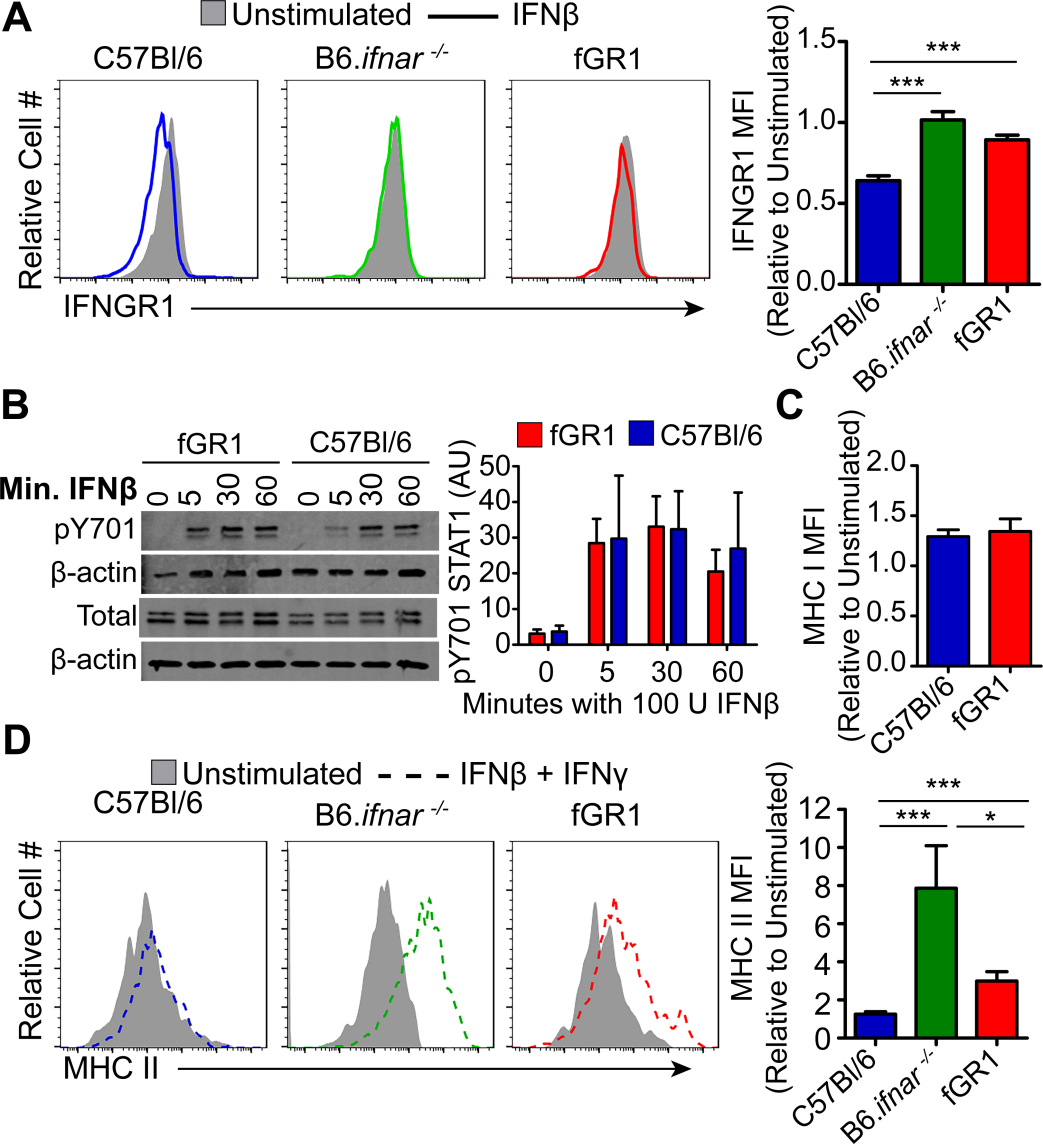
fGR1 macrophages respond to type I IFNs and retain high cell surface IFNGR1 expression. Naïve peritoneal macrophages were treated with 100 U/mL of IFNβ A) for 6 hrs to determine surface IFNGR1 expression; B) 5, 30, 60 min to evaluate STAT1 pY701 by western blot. pSTAT1 bands were normalized to β-actin loading control, Arbitrary Units (AU); C) for 24 hrs and MHC I expression determined by flow cytometry. D) Macrophages were pre-treated with 100 U/mL of IFNβ for 6 hrs before stimulation with 100 U/mL of IFNγ for 24 hrs and MHC II up regulation determined. All MFIs were normalized to their respective unstimulated controls and pooled from multiple experiments (n = 3 independent experiments, 1–3 mice per experiment, *Two-Tailed t-test).

In contrast to our experiments above showing no effects of fGR1 on basal macrophage IFNGR1, fGR1 macrophages were nearly as resistant as B6.*ifnar*^-/-^ macrophages to IFNβ stimulated down regulation of IFNGR1 (Fig 3A). Indeed, IFNGR1 staining in the IFNβ-treated fGR1 macrophages were not significantly lower than unstimulated controls or staining on B6.*ifnar1*^-/-^ macrophages. This was not due to altered sensitivity of fGR1 macrophages to type I IFN. Rather, WT and fGR1 peritoneal macrophages responded similarly to IFNβ stimulation as measured by STAT1 phosphorylation (Y701) at 5, 30, and 60 minutes (Fig 3B), and similar upregulation of MHC I after overnight IFNβ stimulation (Fig 3C).

We next investigated how retaining IFNGR1 affects functional responses in the fGR1 macrophages. Even modest reductions in cell surface IFNGR1 associate with impaired responsiveness to IFNγ and MHC II upregulation [28,32,33]. We thus evaluated upregulation of cell surface MHC II as a sensitive measure of IFNγ responsiveness. Treatment of WT C57Bl/6 but not B6.*ifnar1*^-/-^ peritoneal macrophages with IFNβ significantly impaired upregulation of MHC II in response to IFNγ (Fig 3D), consistent with reductions IFNGR1 (Fig 3A). Induction of MHC II under these circumstances was significantly higher in fGR1 versus WT C57Bl/6 macrophages. This indicates that fGR1 expression overcame the suppressive effects of type I IFN. Nevertheless, IFNβ-treated fGR1 macrophages did not upregulate MHC II as effectively as B6.*ifnar*^-/-^ cells treated with IFNβ and IFNγ (Fig 3D). This difference likely reflects the modest reduction in IFNGR1 observed in the IFNβ-treated fGR1 macrophages (Fig 3A). In conclusion, fGR1 largely (though not completely) restored IFNGR1 expression and responsiveness to IFNγ signaling in macrophages exposed to type I IFN.

### fGR1 mice resist infection by *Listeria monocytogenes*

**Fig 4:**
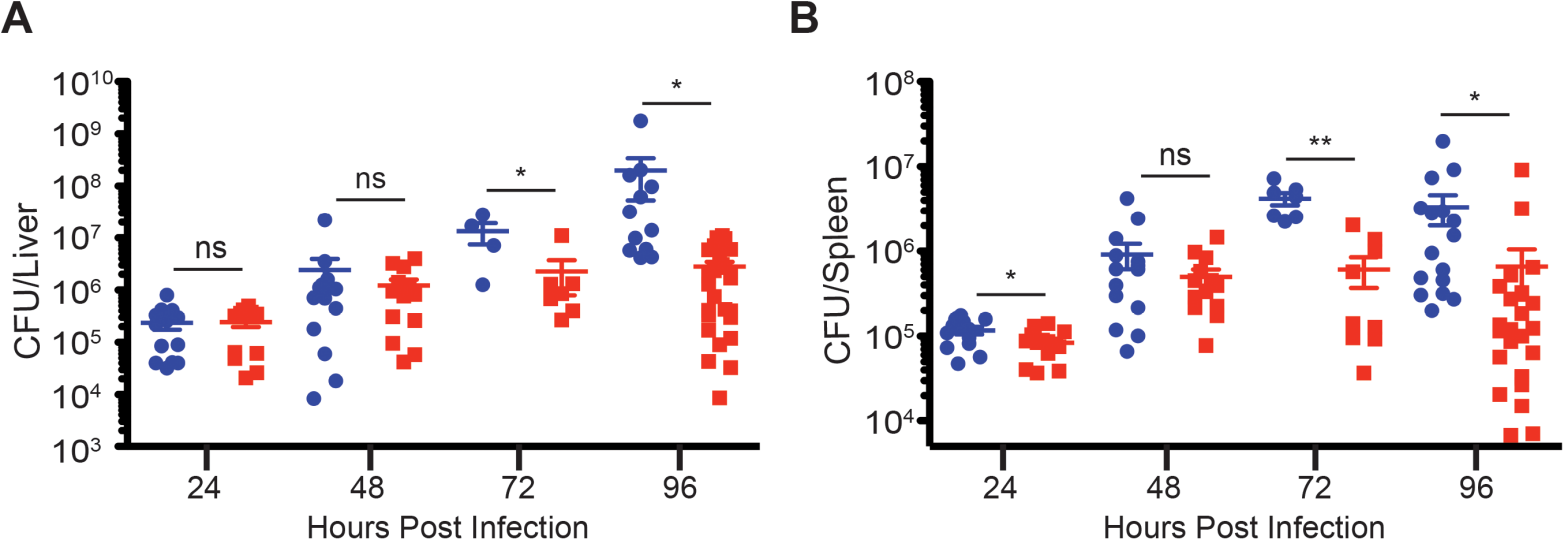
fGR1 mice are more resistant to *L*. *monocytogenes* infection. WT C57Bl/6 (blue) and fGR1 (red) mice were infected intravenously with 1.0 x 10^4^ CFUs of *L*. *monocytogenes*. Bacterial burdens were determined in both the A) liver and B) spleen at 24, 48, 72 and 96 hpi. (All time points are pooled from at least 3 independent experiments, 3–5 mice per group per experiment, *Two-Tailed t-test).

Deficiency in IFNAR1 expression renders mice highly resistant to infection by diverse pathogens, including *L*. *monocytogenes* [20–22,28,34]. The resistance seen in type I IFN-unresponsive mice is characterized by similar *L*. *monocytogenes* burdens early after infection, but 100–1000 fold reductions by 3–4 dpi [20–22,28,34]. To assess how blunting IFNGR1 down regulation might impact bacterial infections, parallel groups of WT and fGR1 mice were inoculated with a sublethal dose of *L*. *monocytogenes*. Bacterial burdens in the livers (Fig 4A) and spleens (Fig 4B) were equivalent in both groups of infected mice through 48 hpi, though the ~1.5 fold reduction in the spleens of fGR1 mice at 24 hpi was significant due to tight grouping of the data. At 72 hpi and beyond, fGR1 mice showed significantly enhanced resistance as illustrated by >70-fold fewer bacteria at 96 hpi (Fig 4A and 4B). Variability in bacterial burdens seen in the control and fGR1 mice likely results from pooling of data from experiments performed over an extended time period. These data indicate that fGR1 mice were consistently and significantly more resistant to systemic *L*. *monocytogenes* infection.

### fGR1 expression does not increase inflammatory myeloid cell accumulation

Type I IFN signaling can stimulate leukocyte apoptosis and correlates with reduced numbers of CD11b^+^ cells in spleens early after *L*. *monocytogenes* infection [20–22]. Consistent with these reports, IL-17, CXCL2, and neutrophil accumulation were modestly increased in tissues of IFNAR1-deficient mice infected with *Francisella tularensis* or *Streptococcus pneumoniae* [17,35]. We thus asked if the enhanced resistance of fGR1 mice to *L*. *monocytogenes* might be associated with increased recruitment of inflammatory myeloid cells to infected tissues. Both CD11b^+^Ly6C^+^Ly6G^lo^ monocytes and CD11b^+^Ly6C^+^Ly6G^hi^ neutrophils accumulated in *L*. *monocytogenes* infected spleens over the first 96 hpi, but the frequency and number of these cells were comparable between WT and fGR1 mice (S2A and S2B Fig). Furthermore, fGR1 expression did not restore the loss of T cells during infection (S2C Fig). These same results were also noted in the liver of WT and fGR1 mice (S3A and S3B Fig). We nonetheless confirmed that numbers of neutrophils, monocytes and T cells in the spleens of infected mice were significantly increased by blockade of IFNAR1 with an anti-IFNAR1 antibody (MAR-1) (S4A and S4B Fig). These data indicate that type I IFNs similarly suppress recruitment or survival of neutrophils, T cells, and inflammatory monocytes in both WT and fGR1 mice. Therefore, restoration of myeloid cell IFNGR1 can increase host resistance even in the presence of these other suppressive effects.

### fGR1 expression permits increased monocyte activation

**Fig 5:**
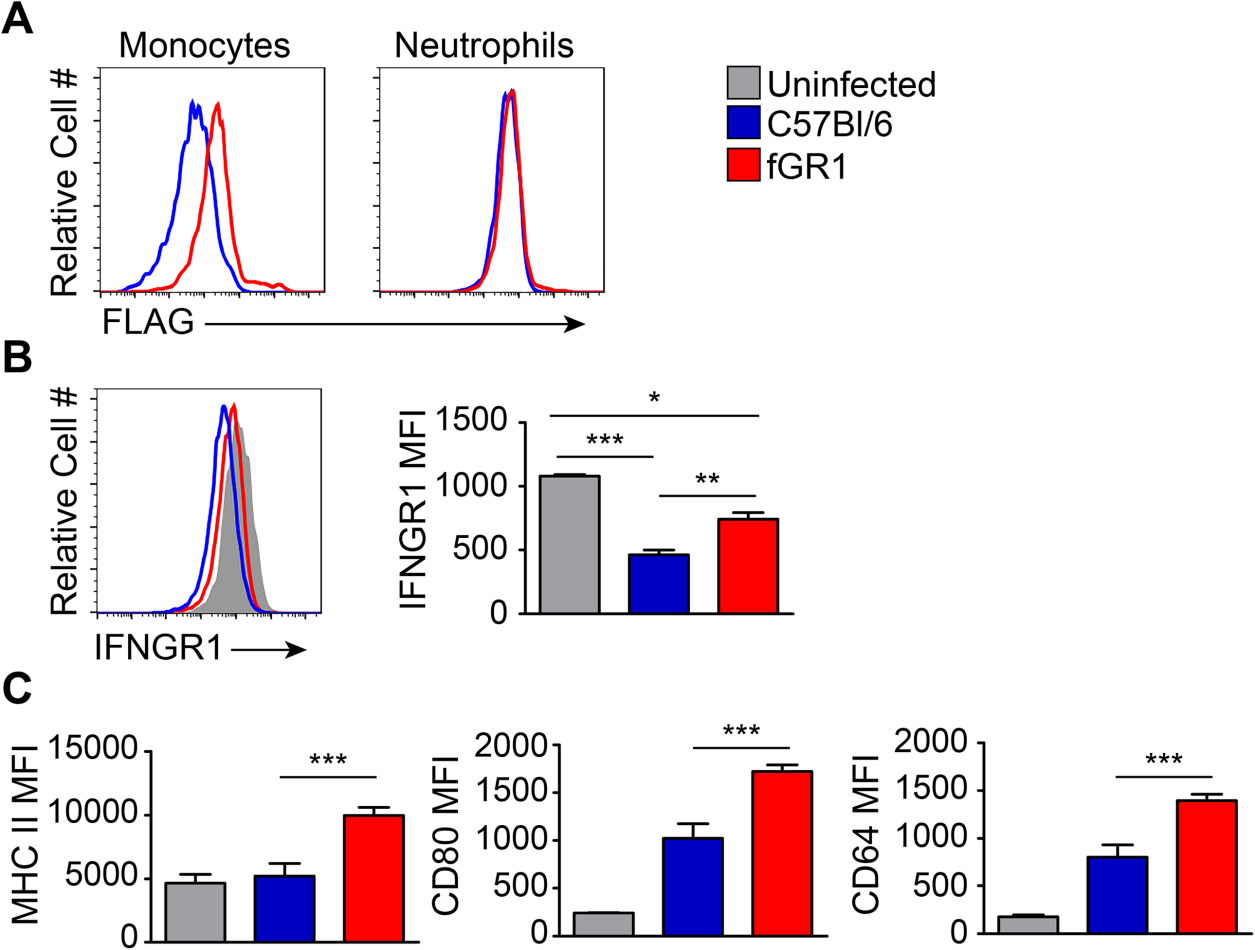
Heightened pro-inflammatory phenotype of fGR1 inflammatory monocytes during *L*. *monocytogenes* infection. Splenocytes from WT (blue) and fGR1 (red) mice 96 hpi *L*. *monocytogenes* infection were analyzed for A) FLAG expression on inflammatory monocytes (CD11b^hi^, Ly6C^hi^, Ly6G^lo^, gated S3A Fig) and neutrophils (CD11b^hi^, Ly6C^hi^, Ly6G^hi^). B) IFNGR1 and C) “M1”-like pro-inflammatory activation markers MHC II, CD80, and CD64 on splenic inflammatory monocytes. (Representative data of one independent experiment, n = 5–10 mice per group. Experiment repeated at least three times. *Two-Tailed t-test).

Anti-FLAG staining was observed on inflammatory monocytes, but not neutrophils, from spleens of *L*. *monocytogenes*-infected fGR1 mice (Fig 5A). Focusing on this population, we evaluated the expression of IFNGR1 and several pro-inflammatory activation markers on monocytes within naïve WT and fGR1 mice and observed no differences (S5A and S5B Fig). However, during infection FLAG staining correlated with an increase in cell surface IFNGR1 on monocytes from the infected fGR1 mice, when compared to those of infected WT C57Bl/6 mice (Fig 5B). Albeit, IFNGR1 staining on fGR1 monocytes was still significantly lower than on monocytes from uninfected spleens (Fig 5B). These results indicate that the low expression of fGR1 enables monocytes to retain higher amounts of IFNGR1 during *L*. *monocytogenes* infection.

IFNγ signaling activates macrophages and monocytes to adopt an “M1” pro-inflammatory and anti-microbial state. This M1 phenotype is associated with increased expression of cell surface markers such as MHC II, CD80, and FcγR1 (CD64). Consistent with their higher IFNGR1 staining, inflammatory monocytes from fGR1 mice had significantly increased cell surface staining for each of these IFNγ-inducible markers (Fig 5C). The increased expression of these markers was not associated with increased production of IFNγ in the *L*. *monocytogenes* infected fGR1 mice (S6 Fig). Rather, these results suggest that the increased retention of cell surface IFNGR1 on monocytes from the fGR1 mice enables them to receive a stronger signal from the IFNγ present.

### Increased resistance of fGR1 mice is dependent on IFNγ signaling

**Fig 6:**
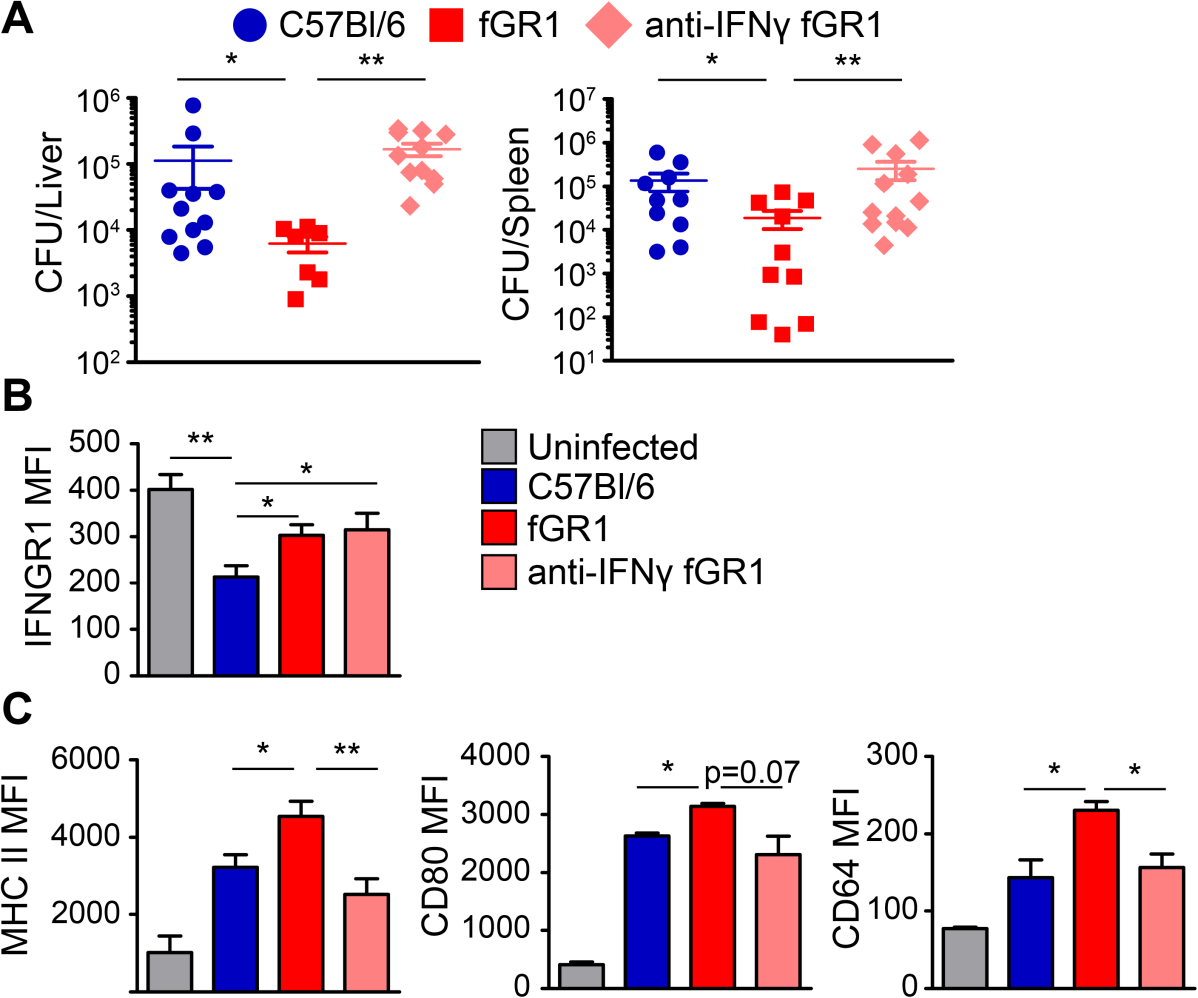
IFNγ is required for increased resistance of fGR1 mice to *L*. *monocytogenes* infection. C57Bl/6 and fGR1 mice were treated with PBS or 500 μg of α-IFNγ depletion antibody 24 hrs before infection with a low dose (5 x 10^3^ CFUs) of *L*. *monocytogenes*. A) Bacterial burdens were determined in both the liver and spleen 72 hpi. (Data pooled from 3 independent experiments, 3–5 mice per group, *Mann-Whitney Test). The splenic inflammatory monocytes were analyzed by flow cytometry for the surface expression of B) IFNGR1 and C) pro-inflammatory activation markers MHC II, CD80, and CD64. (MFIs are pooled from 2-independent experiments, 3–5 mice per group, *Two-Tailed T-test).

To ask more directly if the increased resistance of fGR1 mice was dependent on IFNγ signaling, we pre-treated groups of mice with 500 μg of anti-IFNγ (XMG1.2) to neutralize IFNγ during early stages of *L*. *monocytogenes* infection. Mice were given a lower bacterial dose (5 x 10^3^ CFU) to prevent early deaths. Results showed that neutralizing IFNγ completely abrogated the heightened resistance of the fGR1 mice (Fig 6A). Neutralization of IFNγ did not affect cell surface IFNGR1 staining on monocytes from the fGR1 mice, which remained significantly increased compared to infected WT C57Bl/6 mice (Fig 6B). However, the increased expression of MHC II, CD80, and CD64 seen in IFNγ-replete fGR1 monocytes was abrogated in the IFNγ-depleted fGR1 mice (Fig 6C). These data demonstrate that IFNγ is necessary for the increased resistance to *L*. *monocytogenes* as well as the increased M1 activation of inflammatory monocytes in the fGR1 mice.

### Expression of fGR1 increases cell-intrinsic resistance to *L*. *monocytogenes* infection

**Fig 7:**
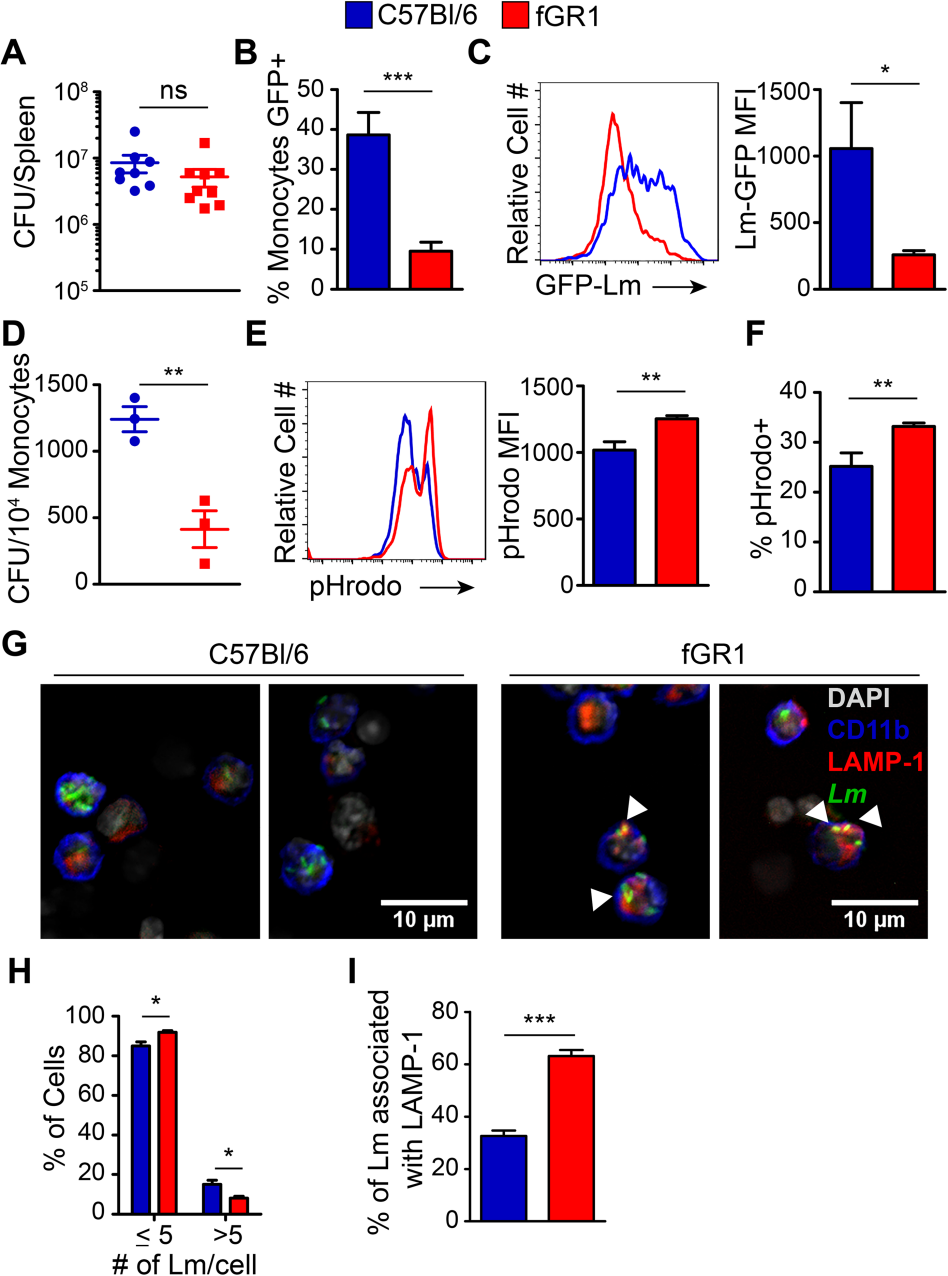
fGR1 expression increases macrophage cell intrinsic ability to kill bacteria. A). Bacterial burdens from C57Bl/6 (blue) and fGR1 (red) mice 60 hpi. Flow cytometry was used to determine the B) frequency of monocytes infected by GFP-expressing *L*. *monocytogenes (Lm)* 60 hpi, and C) MFI of GFP-*Lm* within the inflammatory monocyte population (Data pooled from 3 independent experiments, 3–5 mice per group). D) Inflammatory monocytes were FACS sorted and lysed to determine *Lm* bacterial burdens 60 hpi. (Representative CFUs from 2 independent experiments, 3 mice per groups per experiment). pHrodo *S*. *aureus* Bioparticles were incubated for 30 min with enriched myeloid cells isolated from 60 hpi spleen and the E) MFI and F) frequency of pHrodo positive inflammatory monocytes was determined by flow cytometry (Representative data from 2 independent experiments, 3 mice per group per experiment). G) Spleens were depleted of CD90.2+, IgM+, NK1.1+, Ly6G+ cells and CD11b+ cells were analyzed by fluorescent microscopy. Arrows indicate colocalization of *Lm* and LAMP-1. Unbiased quantification of the H) relative distribution of the number of *Lm* per CD11b+ cell and I) percentage of *Lm* colocalized with LAMP-1 per CD11b+ cell. (Representative data from 2 independent experiments, 3 mice per group, *Two-Tailed T-test).

We next asked if the increased resistance to systemic infection in fGR1 mice was associated with reduced ability of *L*. *monocytogenes* to persist in monocytes with increased IFNGR1 expression and M1 activation. Since bacterial burdens in the WT and fGR1 mice first significantly diverged between 48 and 72 hpi (Fig 4), we examined bioparticle uptake, phagosome maturation, and *L*. *monocytogenes* survival at 60 hpi. Bacterial burdens did not significantly differ between WT and fGR1 mice at this timepoint (Fig 7A), though there was a modest reduction in burdens in the fGR1 spleens. Mice were first inoculated with GFP-expressing *L*. *monocytogenes* and we used flow cytometry to quantify GFP fluorescence in gated inflammatory monocytes. The frequency of monocytes with high amounts of GFP fluorescence was significantly higher in the WT cells (Fig 7B and 7C). We then sorted monocytes and plated lysates to calculate CFU per monocyte. Again, we observed a significant (~3-fold) reduction in the number of live *L*. *monocytogenes* recovered from the fGR1 cells (Fig 7D). Together, these data demonstrate that fewer fGR1 monocytes were productively infected by *L*. *monocytogenes*.

We considered that the reduced frequency of infected fGR1 inflammatory monocytes and the reduced bacterial burdens within these monocytes might reflect defective uptake of bacterial particles. To address this, we used a bead-based approach to enrich for CD11b^+^ cells from spleens of *L*. *monocytogenes*-infected mice (see S7 Fig). The purified cells were then incubated with fluorescence-labeled *Staphylococcus aureus* Bioparticles for 30 min incubation at 37°C. The bioparticles were labeled with a pH-sensitive dye (pHrodo) that only fluoresces at an acidic pH. Hence, we used flow cytometry to evaluate uptake of the particles into acidified compartments in gated inflammatory monocytes. Results indicated that the proportion of fGR1 monocytes staining bright for pHrodo was increased significantly, as was the MFI of pHrodo staining in these cells (Fig 7E and 7F). We interpret these data to indicate that inflammatory monocytes expressing fGR1 have an enhanced ability to phagocytose bacteria into compartments that are maturing. Thus, the reduced frequency of infected inflammatory monocytes in the fGR1 mice was not due to impaired phagocytosis.

The pathogenesis of *L*. *monocytogenes* involves bacterial escape from phagosomal compartments into the cytosol of infected cells, where the bacterium undergoes rapid replication. *L*. *monocytogenes* that are retained in phagosomes are unable to replicate and can be degraded following phagosome fusion with LAMP-1^+^ lysosomes [36,37]. We thus next asked whether reduced bacterial burdens and heightened IFNγ responsiveness seen in fGR1 monocytes during systemic *in vivo* infection might be associated with improved ability of myeloid cells to restrict *L*. *monocytogenes* to phagosomal compartments. Here, we again used beads to negatively enrich myeloid cells (S7 Fig) from spleens of WT and fGR1 transgenic mice at 60 hpi with GFP-expressing *L*. *monocytogenes*. We then assessed the abundance of GFP bacteria and the frequency of their co-localization with the lysosomal marker LAMP-1 using fluorescence microscopy. Staining of the isolated cells revealed the presence of bacteria (green) in CD11b^+^ cells (blue) (Fig 7G). In cells from WT B6 mice, bacteria were rarely observed to co-localize with LAMP-1 (red). This result is consistent with bacterial persistence in immature phagosomes or escape into the cell cytoplasm. By contrast, in cells from infected fGR1 mice bacteria frequently co-localized with LAMP-1 (Fig 7G, right). Using the cell function of the Imaris software suite we unbiasedly quantified these differences. The results showed that more CD11b^+^ cells from the fGR1 mice contained low numbers of *L*. *monocytogenes* bacteria and fewer of these cells contained high numbers of bacteria (Fig 7H). Furthermore, the proportion of bacteria co-localized with LAMP-1 was significantly higher in CD11b^+^ cells from fGR1 mice that contained GFP-expressing *L*. *monocytogenes* (Fig 7I). These data suggest that increased M1 activation enables fGR1 myeloid cells to better engulf and contain *L*. *monocytogenes* within mature phagosomes and phagolysosomes. Hence, despite the continued presence of other responses previously associated with “pro-bacterial” effects of type I IFNs our data demonstrate that attenuating the loss of IFNGR1 suffices to increase myeloid cell-intrinsic bacterial killing and host resistance during systemic *L*. *monocytogenes* infection.

## Discussion

Animal infection studies over the past decade have revealed the ability of type I IFNs to potently increase host susceptibility to diverse bacterial pathogens [3,14]. Gene expression patterns associated with type I IFNs have also been implicated in increased disease severity in humans with naturally-encountered *M*. *tuberculosis* and *Mycobacterium leprae* infections [15,38]. Improved understanding of the mechanisms responsible for the “pro-bacterial” effects of these cytokines thus has relevance to human health. Our studies here used a systemic *L*. *monocytogenes* infection model to provide new experimental evidence in support of the model that type I IFNs exacerbate infection, at least in part, by suppressing macrophage responsiveness to IFNγ. Based on our prior finding that type I IFNs silence *de novo* transcription of *ifngr1* to down regulate myeloid cell expression of IFNGR1 [26,28], we developed a transgenic mouse model to express FLAG-tagged IFNGR1 at low levels using the heterologous *c-fms* promoter. Characterization of these mice confirmed the transgenic “fGR1” receptor is selectively expressed in macrophages at a low level that did not increase overall IFNGR1 and did not alter macrophage responsiveness to IFNγ stimulation in the absence of type I IFNs. Nevertheless, macrophages expressing fGR1 resisted the loss of IFNGR1 that is normally induced by stimulation with type I IFNs and under these conditions showed increased ability to respond to IFNγ. Consistent with these findings, fGR1 mice better resisted systemic *L*. *monocytogenes* infection. The increased resistance of fGR1 mice was also dependent on IFNγ. These data support the model that suppression of macrophage IFNγ responsiveness contributes to type I IFN-dependent increase in susceptibility to *L*. *monocytogenes* and *M*. *leprae* infections [3,15,28]. In addition, our studies here showed that the increased resistance of fGR1 mice correlates with increased expression of IFNGR1 and pro-inflammatory markers by inflammatory monocytes. Further, bacterial loads were reduced in inflammatory monocytes from fGR1 mice and we observed increased association of *L*. *monocytogenes* with the lysosomal marker LAMP-1 in CD11b^+^ cells. These results argue that the increased ability of this myeloid cell population to retain IFNGR1 and respond to IFNγ led to increased activation of cell-intrinsic resistance mechanisms. Overall, our data suggest that blocking the ability of type I IFNs to suppress macrophage IFNGR1 restores host resistance to systemic *L*. *monocytogenes* infection by improving antimicrobial “M1” activation of monocytes recruited to sites of infection.

As mentioned above, diverse other bacterial pathogens also benefit from type I IFN signaling–including *M*. *tuberculosis* [18,39,40]. Patients with untreated pulmonary tuberculosis were reported to have decreased IFNGR1 expression on circulating CD14^+^ monocytes compared to healthy uninfected controls [27]. Furthermore, IFNGR1 expression was restored upon antitubercular treatment in many of these patients [27]. Thus, *M*. *tuberculosis* bacteria might exploit type I IFN-induced suppression of IFNGR1 in myeloid cells. The extent to which this reduction in IFNGR1 is a cause of host susceptibility remains to be explored, however, others have proposed alternative mechanisms that likely also contribute. Specifically, type I IFNs also suppress interleukin-1 and prostaglandin production [23], increase IL-10 production by macrophages [3,14], suppress recruitment of inflammatory neutrophils [17,35], and can promote cellular apoptosis [21,22]. Using antibody blockade of IFNAR signaling we confirmed that responsiveness to type I IFNs corresponds with suppressed recruitment or survival of inflammatory monocytes, neutrophils, and T cells in the spleens of *L*. *monocytogenes*-infected mice. However, the increased resistance seen in fGR1 mice was not associated with increased numbers of T cells or inflammatory monocytes and neutrophils. Thus, blockade of IFNGR1 down regulation appears sufficient to increase host resistance even in the presence of type I IFN-dependent reductions in T cell and inflammatory cell numbers. Previous work in our laboratory has also implicated NK cells as a major source of serum IL-10 detected early after *L*. *monocytogenes* infection [41], and failed to see direct suppression of macrophage cell surface IFNGR1 by IL-10 [28]. These data suggest fGR1 expression and the preservation of IFNGR1 expression enhances macrophage activation independent of effects on IL-10 production. Nevertheless, additional future studies will be necessary to conclusively define the relative impact of these and other processes on type I IFN-driven increases in susceptibility to *M*. *tuberculosis* and other pathogens. The fGR1 mice may also be useful more generally to better define how altering regulation of myeloid cell IFNGR1 affects inflammatory and immune responses in other settings, including viral infections, cancer, and inflammatory/autoimmune diseases.

Increased activation of myeloid cells could potentially improve host resistance by cell intrinsic, cell extrinsic, or both mechanisms. Since fGR1 inflammatory monocytes have increased expression of MHC II and CD80 we considered that the increased resistance of fGR1 mice might be associated with an increased activation of T cells. However, fGR1 expression reduced bacterial burdens within 72–96 hpi, which is prior to peak T cell activation. Instead, we observed that inflammatory macrophages had reduced bacterial burdens and evidence of increased *L*. *monocytogenes* containment within LAMP-1^+^ phagosomes/lysosomes. Early phagosomes are commonly marked by their association with the small GTPase Rab5a which then recruits lysosomal proteins, including LAMP-1, to form a mature phagolysosome [36]. To escape the phagosome, *L*. *monocytogenes* must delay this phagosomal maturation by lysing the vacuole before it associates with impenetrable LAMP-1+ lysosomes. Such escape is a crucial determinant of pathogenicity [37,42,43]. Hence, improved containment of *L*. *monocytogenes* to phagosomes suffices to restrict infection. There is furthermore evidence that IFNγ stimulation of macrophages improves their ability to mediate such containment [44]. How IFNγ mediates this effect is not clear. IFNγ can modulate phagosome maturation by increasing Rab5a function [45], but also upregulates expression of NADPH oxidase subunits [7] and guanosine triphosphatase (GTPases). The latter are known to associate with bacteria-containing phagosomes [46], and to alter vesicular trafficking and phagosomal maturation [47]. Additional studies are needed to distinguish the relative importance of these various mechanisms in mediating the resistance of fGR1 macrophages. Future studies comparing WT and fGR1 macrophages could identify additional mechanisms through which IFNγ signaling can increase macrophage resistance to *L*. *monocytogenes* infection.

Humans are thought to encounter *L*. *monocytogenes* infection through ingestion of contaminated foods and clinical disease in humans is typically associated with bacteremia or meningitis. However, the impact of type I IFNs on human Listeriosis remains unclear. Mice are not highly susceptible to development of high-titer systemic *L*. *monocytogenes* infections following oral inoculation [48]. In an effort to create a better oral infection model, a *L*. *monocytogenes* strain was engineered to express a mutant InlA that binds more avidly to mouse E-cadherin [49]. *L*. *monocytogenes* expressing this “murinized” InlA (InlA^m^) show increased invasiveness for epithelial and certain non-epithelial cell types [50], but they still fail to achieve high-titer systemic infection [51]. Using *L*. *monocytogenes* that express InlA^m^, two recent papers presented data suggesting *ifnar1* deficiency fails to substantially increase resistance during the low-burden oral *L*. *monocytogenes* infection [51,52]. Low systemic bacterial burdens were correlated with low systemic concentrations of type I IFNs in these mice [51,52]. Because both IFN production and bacterial burdens were low, it is thus unclear how to interpret the fact that *ifnar1*-deficient mice did not have further reduced burdens in these studies. Low type I IFN production may have either caused or been the result of the low systemic bacterial burdens seen in these studies. Thus, while it remains to be seen how relevant the study of systemic *L*. *monocytogenes* infection is for understanding of human Listeriosis, it also remains unclear if the mouse oral infection model accurately reflects human disease. Given this and the fact that type I IFNs increase susceptibility to other bacterial infections through mucosal and non-mucosal epithelia colonized by microbes, such as the lung [18,23,25,40,53], skin [15], and urogenital tract [54], we argue that it remains worthwhile to use the systemic *L*. *monocytogenes* infection model in studies of the mechanisms responsible for the pro-bacterial effects of these cytokines–as well as in studies dissecting other aspects of host/pathogen biology.

In conclusion, our studies provide evidence that host type I IFNs increase susceptibility to systemic *L*. *monocytogenes* bacterial infection by promoting loss of IFNGR1 on myeloid cells. While not addressed in our work, it is plausible that this process may also be a contributing factor towards host susceptibility in other bacterial infections. Drugs that target and block this process may thus prove to be useful in the clinical management of severe disseminated Listeriosis and other systemic and mucosal bacterial infections. The development and spread of antibiotic resistance mechanisms is a worldwide problem that limits clinical options for treatment of common infections [55]. New therapies for treating bacterial infections are thus urgently needed. Development of host-directed therapeutics that target and inhibit deleterious effects of host type I IFNs appears to be an attractive approach as such host-directed therapies may fail to drive the continued development and dissemination of antibiotic resistance mechanisms and thus have potential for prolonged life in the clinic.

## Methods

### Mice

fGR1 transgenic mice were created by inserting IFNGR1 cDNA into BsrGI and AgeI restriction digest sites of the 7.2 fms-EGFP transgenic vector kindly provided by Dr. David A Hume [30]. DNA encoding a single FLAG-epitope (DYKDDDDK) was placed after a signal sequence between residues 6 (glutamic acid, E) and 7 (aspartic acid, D) in the N-terminal extracellular domain of the endogenous receptor [56]. Cleavage of the endogenous signal peptide is predicted to generate a mature fGR1 protein with the N-terminal sequence EDYKDDDDKD and not interfere with IFNγ binding. The plasmid was linearized and microinjected into C57Bl/6J blastocysts by the National Jewish Health transgenic mouse core facility. Mice were screened using primers specific for the transgene construct: 5’-GGAGGCGCCCACGTAGGTC and 5’-AGCTTTAACTCTGGCCCAGGC. All fGR1 transgenic mice used in these studies originated from a single founder. For experiments in Fig 1C, fGR1 transgenic mice were crossed onto the B6.*ifngr1*^-/-^ background such that myeloid cells express only fGR1. WT C57Bl/6J control mice and B6.*ifngr1*^-/-^ mice were purchased through Jackson Laboratories and maintained in our specific pathogen free (SPF) colonies at National Jewish Health and the University of Colorado medical campus. B6.*ifnar1*^-/-^ were described previously [28], and maintained in our SPF facilities.

### Organ processing for single-cell suspension

Spleens were harvested into media containing 1% penicillin/streptomycin then transferred to a solution of 1 mg/mL of collagenase type IV (Worthington) in HBSS plus cations (Gibco). After a 25-min incubation at 37°C, spleens were passed through a 70 μM cell strainer and the cell suspension was treated with RBC lysis buffer (0.15 M NH_4_Cl, 10mM KHCO_3_, 0.1 mM Na_2_EDTA, pH 7.4) for 3 mins. Similar to splenic preparations, livers were also harvested into penicillin/streptomycin containing media then transferred to a solution of 1 mg/mL of collagenase IV. After 30 mins at 37°C, livers were disrupted over a 70 μM cell strainer and the cells were re-suspended in 40% Percoll (GE Healthcare). The 40% Percoll was underlayed with 60% Percoll, and after centrifugation liver cells were collected from the gradient interface. Red blood cells were lysed with RBC lysis buffer for 1–3 min. Blood was collected via submandibular or cardiac puncture and harvested into HBSS without cations and heparin (Sigma). Cells were exposed to RBC lysis buffer for 1–3 min twice to eliminate red blood cells. Lungs were perfused with 10-mL ice cold PBS then harvested and processed the same as the spleen. The femurs were harvest and flushed with penicillin/streptomycin containing media to collect the bone marrow. The cell suspension was treated with RBC lysis buffer for 3 min. Peritoneal cells were harvested by injecting 10-mL ice cold PBS into the peritoneal cavity. Resected intestines were washed with ice-cold PBS to remove fecal content. The tissue was then cut longitudinally and minced into small 3–5 mm pieces. The intestinal pieces were vigorously vortexed in IEL solution (HBSS minus cations, 15mM HEPES, 1 mM EDTA) for 5 mins at least three times to remove mucus and epithelial cells. Tissues were then washed with ice-cold PBS and transferred into a LPL digestion solution containing 150 U/mL collagenase VIII (Sigma), 10% FBS, 15mM HEPES, and 1% penicillin/streptomycin for 15–60 min at 37°C with vigorous shaking.

### Cell culture and cytokine stimulations

Peritoneal cells were isolated from naïve mice by lavage using 10 mL of ice cold PBS from the peritoneal cavity. Non-tissue culture treated suspension plates were used to minimize cell adherence for experiments involving flow cytometric surface expression analysis. For experiments using western blot analysis, peritoneal cells were placed on tissue-culture treated plates for several hours to ensure adherence by the macrophages and non-adherent cells were removed by vigorous washes with PBS. Cells were cultured in DM10 media (DMEM supplemented with 10% FBS, 1% sodium pyruvate, 1% L-glutamine, 1% penicillin/streptomycin). In experiments evaluating IFNGR1, cells were treated with 100 Units (U)/mL murine IFNβ (PBL, #12401–1) for 6–8 hrs. For experiments evaluating MHC II up regulation cells were treated ± 100 U/mL IFNβ for 6–8 hrs followed by treatment with 100 U/mL murine IFNγ (LifeTechnology, #PMC-4031) for 18–24 hrs. For MHC I expression, cells were treated with 100 U/mL IFNβ for 24 hrs.

### Flow cytometry and FACS sorting

Murine Fc receptors were blocked before staining using supernatant from hybridoma 2.4G2 (rat anti-CD16/32). The following mAbs were diluted in FACS buffer (1% BSA, 0.01% NaN3, PBS): CD11b (M1/70, eBioscience), CD11c (N418, Biolegend), Ly6C (Hk1.4, eBioscience), Ly6G (1A8, BioLegend), CD90.2 (53–2.1, eBioscience), CD64 (X54/5/7.1, BioLegend), CD80 (16-10A1, eBioscience), IgM (II/4I, eBioscience), MHC II (I-A/I-E) (M5/114.15.2, eBioscience), F480 (CL-A3-1, BioRad), IFNGR1/CD119 (2E2, BD Bioscience), MHC I (H-2Db) (28-14-8, eBioscience). For FLAG-tag staining experiments either used mouse α-DYKDDDDK IgG-PE (M2, Columbia Bioscience) or a custom Ab to fGR1 (BioMatik) that was biotinylated (Pierce). FLAG-tag or IFNGR1 (CD119) were stained using a secondary Streptavidin-APC (eBioscience). Cells were analyzed on either a BD LSR II or BD LSR Fortessa (BD Biosciences) and data were processed with FlowJo software (Treestar). For FACS sorting, splenocytes were processed as described above and enriched for myeloid cell populations by depleting lymphocytes. To deplete, splenocytes were incubated with PE-tagged antibodies (specific for CD90.2, IgM, and NK1.1) and α-PE Microbeads (Miltenyi Biotec). Cell suspensions were added to LS magnetic columns (Miltenyi Biotec) and the flow-through collected and stained for FACS cell sorting. At least 1 x 10^5^ cells were sorted on BD Aura Fusion with a purity of >90%. For determining CFUs from sorted populations, cells were lysed in 0.02% NP-40 and serial dilutions plated on Tryptic Soy Broth (TSB) agar plates with 50 mg/mL Streptomycin. Total CFUs were counted and normalized to the exact number of cells sorted and lysed.

### Western blots

Naïve peritoneal macrophages were treated with indicated concentrations of IFNγ or IFNβ for either 0, 5, 30, 60 minutes. Cells were lysed in 0.02% NP-40 supplemented with HALT protease and phosphatase inhibitors (Thermo Scientific) directly in the tissue culture dish. Protein concentrations were determined by BCA protein assay (Pierce) and 1x SDS-PAGE buffer (0.0625 M Tris-Cl, pH 6.8/2% SDS/10% glycerol/5% 2-ME/0.01% Bromophenol Blue) was added. Equivalent protein amounts were loaded into 10% acrylamide gels and transferred onto PDVF membranes (Millipore). Blots were probed for pY701 STAT1 (58D6, Cell Signaling) or Total STAT1 (91-C, Cell Signaling) with β-Actin (8H10D10, Cell signaling) as a loading control on each blot. Blots were developed using the secondary antibodies, goat α-rabbit IR 800 (926–32211, LI-COR) and goat α-mouse IR 680 (926–68070, LI-COR), and imaged on an Odyssey CLX (LI-COR). All pSTAT1 bands were normalized to β-actin on the same blot using ImageStudio Ver 4.0 software (LI-COR). Densitometry graphs are pooled from at least 3 independent pSTAT1 blots.

### Bacterial infections

Male and female gender-matched 8–12 week mice were infected with WT mouse-passaged 10403s *L*. *monocytogenes* (Lm) strain. For most experiments, a sub-lethal dose of 1–1.5 x10^4^ CFUs/mouse was given via tail vein i.v. inoculation. Lm from thawed aliquots was grown to log phase in TSB media prior to infection. Spleen and liver were harvested at 24, 48, 72, or 96 hrs post infection (hpi) to quantify bacterial burdens and assess cell populations by flow cytometry. To quantify cytokine production, serum was obtained from clotted blood collected via cardiac puncture and was stored at -20°C until use. Serum was diluted at least 1:2 and IFNγ cytokine production measured by ELISA (BD Biosciences). Bacterial burdens were determined as previously described [28,41,57]. For GFP-Lm infections, a WT 10403s Lm strain expressing a GFP plasmid with Erm resistance was injected at a high dose of 2 x 10^5^ CFUs/mouse for 60 hrs. For cytokine depletion experiments, PBS, 0.5 mg of α-IFNγ Ab (XMG1.2, BioXcell), or 0.5 mg α-IFNAR1 (MAR-1, BioXcell), was injected intraperitoneally (I.P.) 24 hrs prior to Lm infection. A lower dose of 5 x 10^3^ CFUs/mouse was used when IFNγ was depleted.

### Phagocytosis assay

Splenocytes from 60 hpi mice were isolated and enriched for myeloid cells by FACS staining with PE-conjugated antibodies specific for CD90.2, IgM, ad NK1.1 then incubating with α-PE beads (Miltenyi Biotec) to deplete lymphocytes as described above. The flow-through from the LS magnetic column (Miltenyi Biotec) was collected and 5 x 10^5^ myeloid cells were plated in a 24-well plate (Gibco) and allowed to adhere for at least 3 hrs. pHrodo *Staphylococcus aureus* Bioparticles (ThermoFisher) were reconstituted as per manufacturer’s instruction in Phagocytosis Buffer (HBSS without cations, 20 mM HEPES, 5 mM EDTA, pH 7.4) to a concentration of 1 mg/mL. The Bioparticles were added to the myeloid cells at a concentration of 30 Bioparticles per cell and incubated at 37°C (experimental) or 4°C (control) for 30 min. Phagocytosis was stopped by the addition of ice-cold Phagocytosis Buffer and the cells were placed on ice. Samples were washed twice in ice-cold Phagocytosis Buffer and once in ice-cold FACS Buffer and then fixed in 1% PFA for 30 min in the dark at room temperature before being stained for flow cytometric analysis.

### Fluorescent microscopy

C57Bl/6 and fGR1 mice were infected with a high-dose of 2–3 x 10^4^ CFUs/mouse of WT (non-GFP) Lm. Single cell suspensions were incubated with PE-tagged antibodies specific for CD90.2, NK1.1, IgM, and Ly6G for depletion using α-PE Microbeads and LS columns (Miltenyi Biotec). The flow-through was collected and largely comprised monocytes and macrophages (S7 Fig). These were stained for surface CD11b (APC), fixed in 4% PFA, and permeabilized with saponin to stain intracellular Lm using Listeria-O rabbit antiserum (Difco) and a F(ab’)2 Anti-Rabbit IgG-FITC secondary (eBioscience). Additional stains were LAMP-1/CD107a- PE (1D4B, eBioscience), and DAPI. The cells were mounted on charged slides by cytospin and images captured within 48 hrs of staining. Images were analyzed using the cell function in Imaris software (Bitplane). The cell body and nuclei were detected by CD11b and DAPI staining, respectively, and Lm was detected on a per cell basis by using the vesicle function. Consistent parameters were applied to all images for unbiased Lm and LAMP-1 detection. Co-localization between these two parameters was determined for each Lm (vesicle) on a per cell basis by applying a consistent positive threshold for the mean intensity of LAMP-1 at each Lm vesicle.

### Statistical analysis

All experiments were repeated at least three times unless otherwise noted. *In vitro* Mean Fluorescent Intensity (MFI) were normalized as previously described [26] to facilitate analysis of data pooled from independent experiments. *In vivo* MFIs are representative figures with at least 3 mice per group, except GFP MFIs which were pooled. Statistical methods were performed using GraphPad Prism software. Significance was determined by paired two-tailed t-test unless otherwise noted. A *p*-value of < 0.05 was considered significant.

### Ethics statement

These studies were conducted with approval by the Animal Care and Use Committees for both National Jewish Health protocol #AS2682-08-16 and the University of Colorado School of Medicine protocol # B-105614(05)1E. These protocols follow standards enacted by the United States Public Health Service and Department of Agriculture.

## Supporting information

S1 Table Leukocyte frequencies and monocyte activation in naïve fGR1 mice. A) The frequencies of leukocyte populations were determined within different organs. Monocytes (CD90.2-, CD11b^hi^, Ly6C^hi^, Ly6G^lo^), Neutrophils (CD90.2-, CD11b^hi^, Ly6C^hi^, Ly6G^hi^), Dendritic cells (CD90.2-, CD11c^hi^, MHC II^hi^), T cells (CD90.2+), and B cells (CD90.2-, IgM+, MHC II^hi^). The average frequency of each population within the Live gate is shown ± the standard deviation. B) The average geometric mean of MHC II on the monocyte population within different organs, ± the standard deviation. (Data pooled from at least 2 independent experiments, 2–3 mice per group per experiment)
(PDF)

S1 Fig fGR1 expression is restricted to monocyte and macrophage populations. FLAG staining on monocytes (CD90.2-, CD11b^hi^, Ly6C^hi^, Ly6G^lo^), macrophages (CD90.2-, CD11b+, F480^hi^), neutrophils (CD90.2-, CD11b^hi^, Ly6C^hi^, Ly6G^hi^), dendritic cells (DC) (CD90.2-, CD11c^hi^, MHC II^hi^), B cells (CD90.2-, IgM+, MHC II^hi^) and T cells (CD90.2+) within the A) peritoneum, B) spleen, C) liver, D) lung (macrophages are CD11c+, F480^hi^ and DCs are CD11b+, MHC II^hi^), E) blood, and F) bone marrow of naïve WT C57Bl/6 (blue) and fGR1(red) mice. (Representative histograms from at least 2 independent experiments.)
(TIF)

S2 Fig Expression of fGR1 does not alter recruitment of splenic inflammatory cells following *L*. *monocytogenes* infection. A) Representative flow plots demonstrate the gating strategy and frequency of both inflammatory monocyte (CD11b^hi^, Ly6C^hi^, Ly6G^lo^) and neutrophil (CD11b^hi^, Ly6C^hi^, Ly6G^hi^) populations at 24 and 96 hpi. B) The total numbers of inflammatory myeloid cells and C) T cell (CD90.2+) populations within the spleen over the course of *L*. *monocytogenes* infection. (All time points are pooled from at least 3 independent experiments, 3–5 mice per group per experiment).
(TIF)

S3 Fig fGR1 expression has no effect on inflammatory cell accumulation in the liver following *L*. *monocytogenes* infection. A) The total number of liver cells collected from 40:60 Percoll gradient. B) The total numbers of inflammatory immune cells in the liver 72 hpi. (Data are pooled from 2 independent experiments, 3 mice per group per experiment).
(TIF)

S4 Fig Blockade of type I IFN signaling increases splenocytes during *L*. *monocytogenes* infection. WT C57Bl/6 mice were treated with PBS or 0.5 mg MAR-1 antibody 24 hrs before *L*. *monocytogenes* infections. The total number of inflammatory monocytes, neutrophils, and T cells was determined by flow cytometry A) 24 or B) 96 hpi. (Both time points are pooled from 2 independent experiments, 2–4 mice per group per experiment, *Two-Tailed T-test).
(TIF)

S5 Fig Naïve fGR1 inflammatory monocytes have similar expression IFNGR1 and pro-inflammatory activation markers. Splenic inflammatory monocytes (CD11b^hi^, Ly6C^hi^, Ly6G^lo^) from uninfected WT C57Bl/6 (blue) and fGR1 (red) were analyzed by flow cytometry for expression of A) IFNGR1 and B) the IFNγ-activated genes MHC II, CD80, and CD64. (Data were pooled from at least 3 independent experiments, 2–3 mice per group per experiment).
(TIF)

S6 Fig IFNγ production is similar in both WT and fGR1 mice in response to *L*. *monocytogenes*. Serum was collected from WT C57Bl/6 (blue) and fGR1 (red) mice 24, 48, 72 and 96 hpi and used in an ELISA to determine the pg/mL of IFNγ. (All time points were pooled from at least 3 independent experiments, 3–5 mice per group per time point).
(TIF)

S7 Fig The composition of myeloid cell enrichment after α-PE bead depletion. Splenocytes 60 hpi with *L*. *monocytogenes* were FACS stained with PE-congregated mAbs for CD90.2, IgM, NK1.1, and Ly6G and incubated with α-PE beads. The composition of cells was determined by flow cytometry. A) Cell populations before LS columns. B) Cells that flowed-through the columns and were collected for analyses in Fig 7. C) The purified population of cells from (B) were stained with DAPI, anti-CD11b, and anti-Lm ± anti-LAMP-1 to evaluate “carry-over” of the PE. Flow plots and images are representative of at least 3 independent experiments with 3 mice per experiment.
(TIF)
