# Supplementary material for: Down regulation of macrophage IFNGR1 exacerbates systemic L. monocytogenes infection
Source: PLoS Pathog. 2017 May 22;13(5):e1006388. doi: 10.1371/journal.ppat.1006388 (PMC5457163; doi:10.1371/journal.ppat.1006388)
Supplement: S1 Table — A) The frequencies of leukocyte populations were determined within different organs. Monocytes (CD90.2-, CD11bhi, Ly6Chi, Ly6Glo), Neutrophils (CD90.2-, CD11bhi, Ly6Chi, Ly6Ghi), Dendritic cells (CD90.2-, CD11chi, MHC IIhi), T cells (CD90.2+), and B cells (CD90.2-, IgM+, MHC IIhi). The average frequency of each population within the Live gate is shown ± the standard deviation. B) The average geometric mean of MHC II on the monocyte population within different organs, ± the standard deviation. (Data pooled from at least 2 independent experiments, 2–3 mice per group per experiment) (PDF) [file ppat.1006388.s001.pdf]

S1A Table. Frequency of leukocyte populations in naïve fGR1 transgenic mice.

	Monocytes		Neutrophil		Dendritic Cells		T cells		B cells	
	C57Bl/6	fGR1	C57Bl/6	fGR1	C57Bl/6	fGR1	C57Bl/6	fGR1	C57Bl/6	fGR1
Spleen	0.909 ± 0.428	1.060 ± 0.257	0.315 ± 0.029	0.404 ± 0.088	0.938 ± 0.157	0.966 ± 0.318	23.37 ± 3.720	24.54 ± 3.760	33.62 ± 5.790	27.82 ± 3.490
Peritoneum	2.033 ± 0.620	1.887 ± 0.656	0.206 ± 0.046	0.289 ± 0.141	2.267 ± 0.942	2.341 ± 1.686	16.34 ± 5.956	21.64 ± 5.686	19.86 ± 4.053	19.35 ± 3.073
Liver	0.756 ± 0.138	0.973 ± 0.296	0.186 ± 0.060	0.179 ± 0.087	1.992 ± 0.702	2.243 ± 1.012	41.08 ± 6.746	38.27 ± 8.383	14.27 ± 3.530	11.38 ± 2.649
Lung	0.848 ± 0.463	0.881 ± 0.436	1.537 ± 1.001	1.185 ± 0.799	1.887 ± 1.131	1.015 ± 0.745	13.79 ± 4.430	10.92 ± 3.450	4.539 ± 3.403	2.713 ± 2.368
Colon	0.298 ± 0.053	0.294 ± 0.158	0.021 ± 0.008	0.063 ± 0.036	nd	nd	1.082 ± 0.384	0.922 ± 0.387	nd	nd

nd = not determined
In all cases, differences between groups were p>0.05 using the Two-Tail T-test

S1B Table. Activation of monocyte populations in naïve fGR1 mice.

	MHC II MFI	
	C57Bl/6	fGR1
Spleen	276.5 ± 86.91	378.3 ± 134.2
Peritoneum	2042 ± 442.2	2542 ± 605.7
Liver	605.3 ± 89.48	580.0 ± 95.16
Lung	357.8 ± 172.6	295.0 ± 148.0
Colon	8598 ± 1386	8853 ± 2029

No significant differences between groups. p>0.05 using the Two-Tail T-test
