# Supplementary figures and images for: Down regulation of macrophage IFNGR1 exacerbates systemic L. monocytogenes infection
Source: PLoS Pathog. 2017 May 22;13(5):e1006388. doi: 10.1371/journal.ppat.1006388 (PMC5457163; doi:10.1371/journal.ppat.1006388)

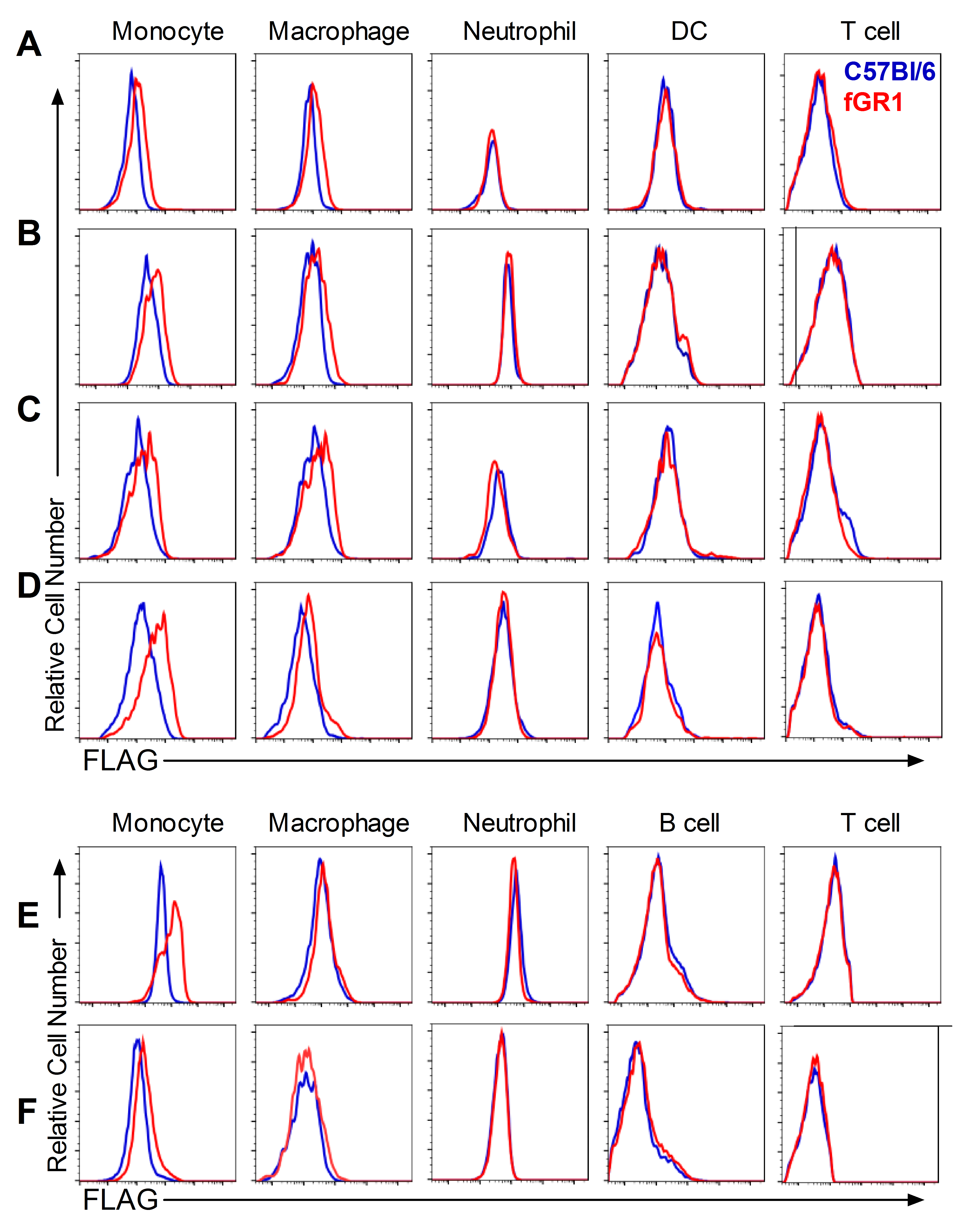

Supplement: S1 Fig — FLAG staining on monocytes (CD90.2-, CD11bhi, Ly6Chi, Ly6Glo), macrophages (CD90.2-, CD11b+, F480hi), neutrophils (CD90.2-, CD11bhi, Ly6Chi, Ly6Ghi), dendritic cells (DC) (CD90.2-, CD11chi, MHC IIhi), B cells (CD90.2-, IgM+, MHC IIhi) and T cells (CD90.2+) within the A) peritoneum, B) spleen, C) liver, D) lung (macrophages are CD11c+, F480hi and DCs are CD11b+, MHC IIhi), E) blood, and F) bone marrow of naïve WT C57Bl/6 (blue) and fGR1(red) mice. (Representative histograms from at least 2 independent experiments.) (TIF) [file ppat.1006388.s002.tif]

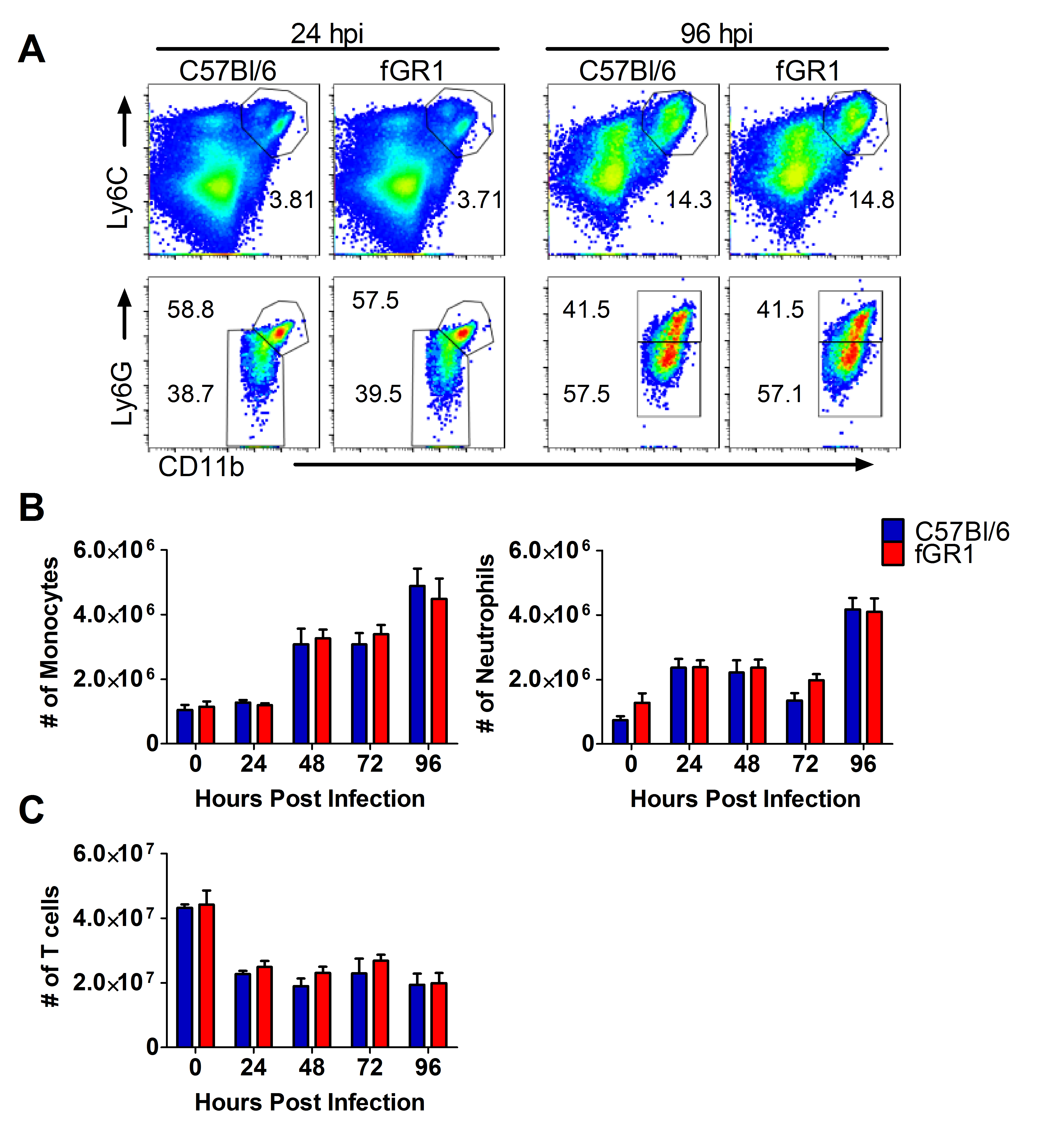

Supplement: S2 Fig — A) Representative flow plots demonstrate the gating strategy and frequency of both inflammatory monocyte (CD11bhi, Ly6Chi, Ly6Glo) and neutrophil (CD11bhi, Ly6Chi, Ly6Ghi) populations at 24 and 96 hpi. B) The total numbers of inflammatory myeloid cells and C) T cell (CD90.2+) populations within the spleen over the course of L. monocytogenes infection. (All time points are pooled from at least 3 independent experiments, 3–5 mice per group per experiment). (TIF) [file ppat.1006388.s003.tif]

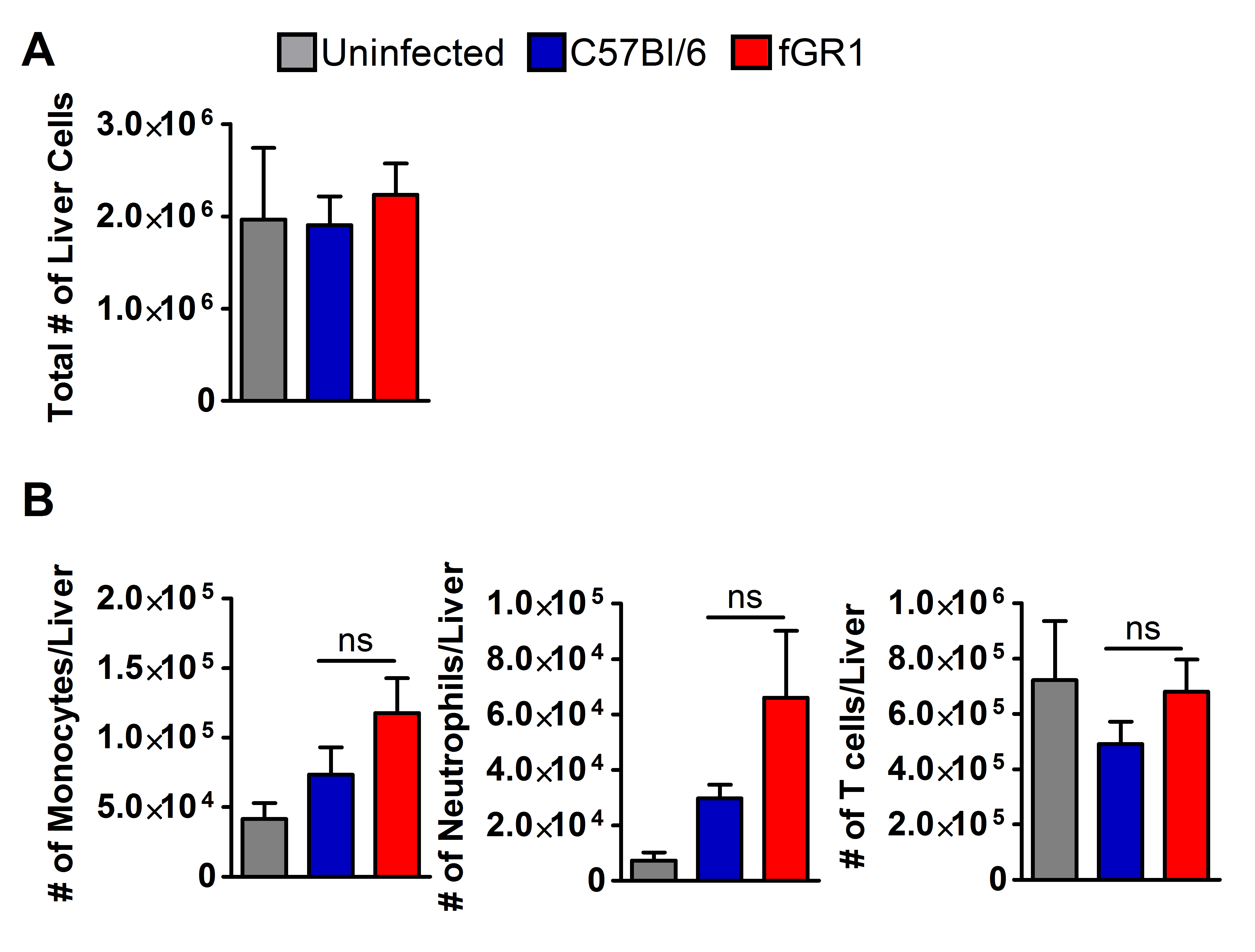

Supplement: S3 Fig — A) The total number of liver cells collected from 40:60 Percoll gradient. B) The total numbers of inflammatory immune cells in the liver 72 hpi. (Data are pooled from 2 independent experiments, 3 mice per group per experiment). (TIF) [file ppat.1006388.s004.tif]

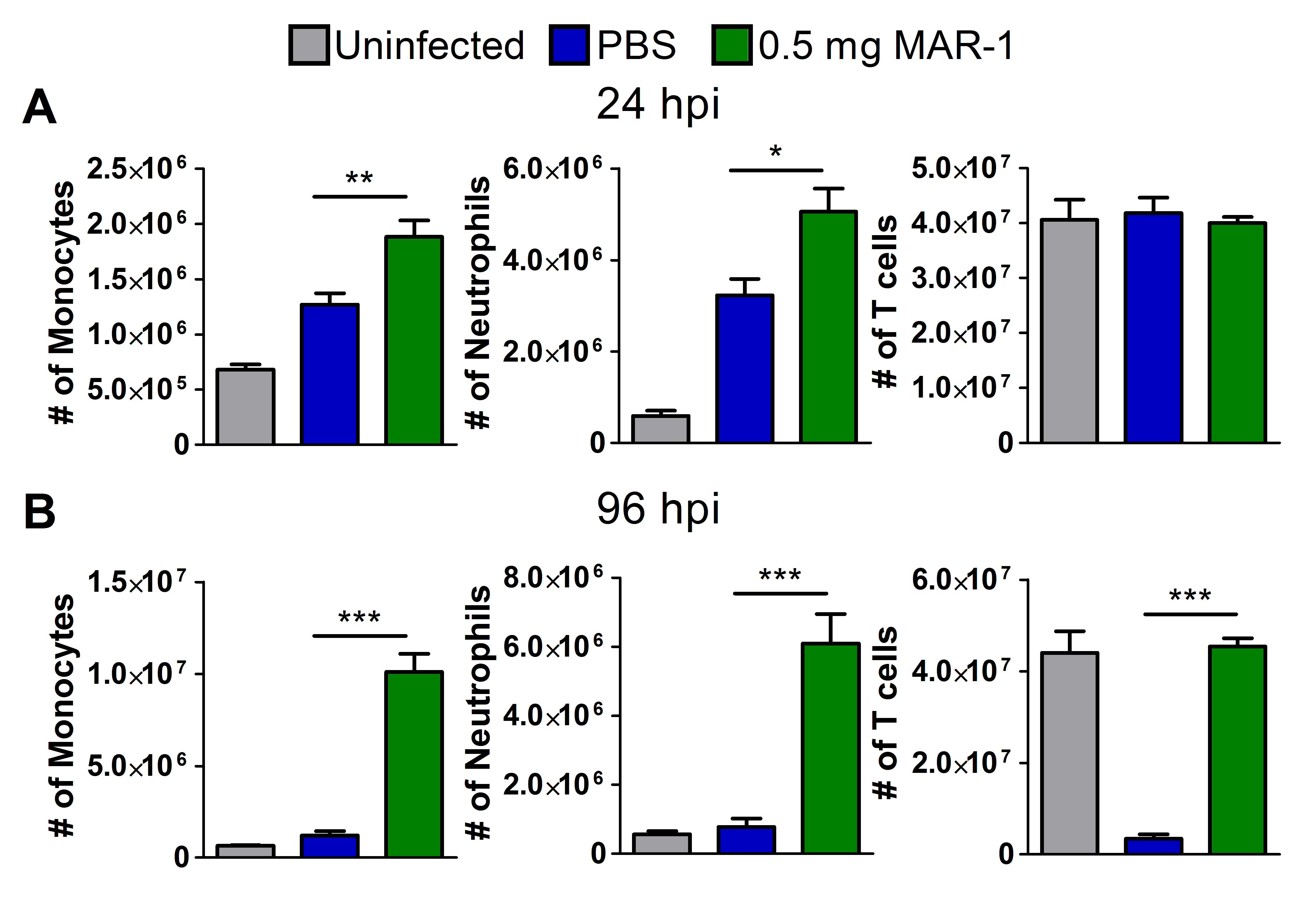

Supplement: S4 Fig — WT C57Bl/6 mice were treated with PBS or 0.5 mg MAR-1 antibody 24 hrs before L. monocytogenes infections. The total number of inflammatory monocytes, neutrophils, and T cells was determined by flow cytometry A) 24 or B) 96 hpi. (Both time points are pooled from 2 independent experiments, 2–4 mice per group per experiment, *Two-Tailed T-test). (TIF) [file ppat.1006388.s005.tif]

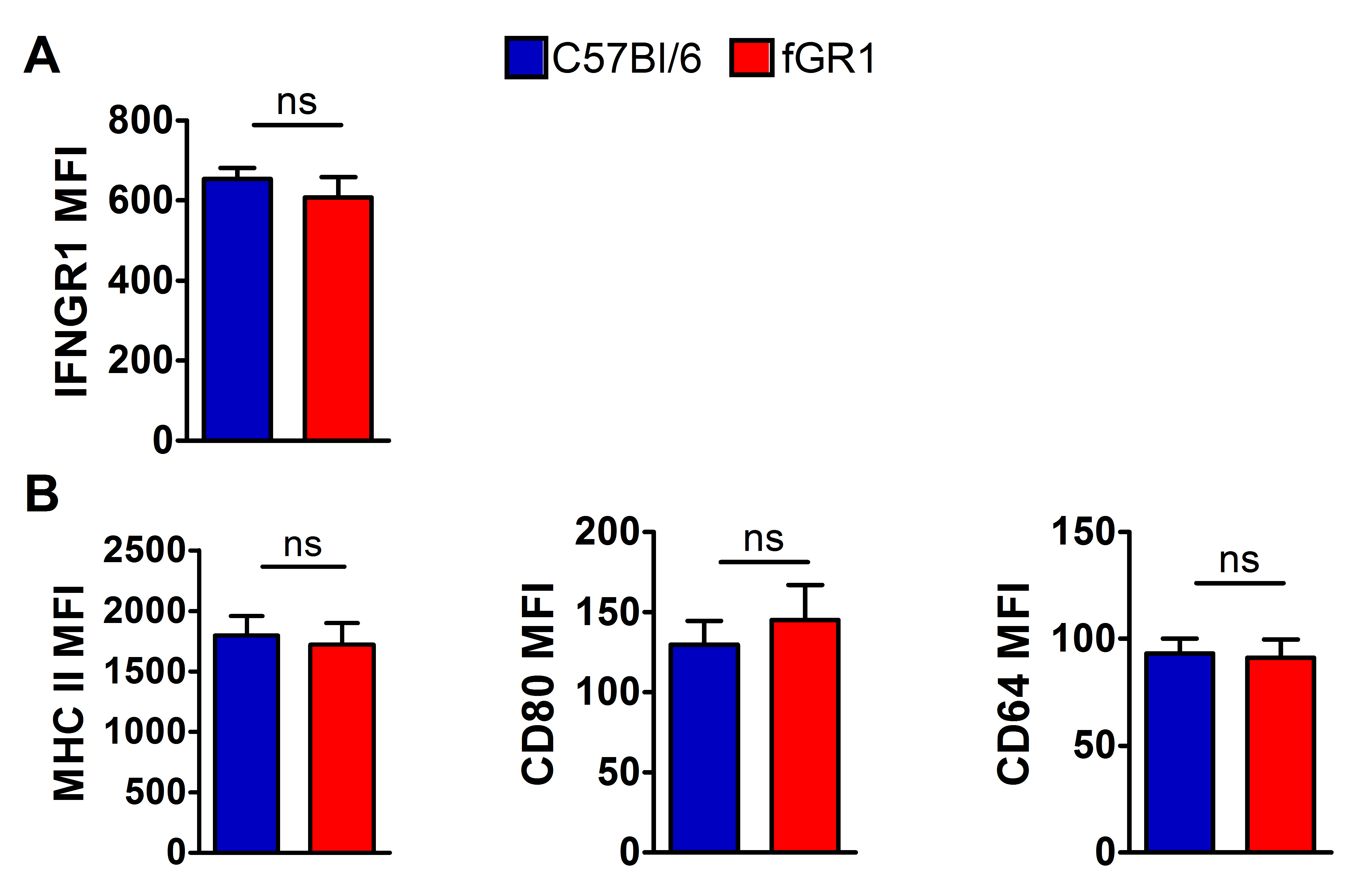

Supplement: S5 Fig — Splenic inflammatory monocytes (CD11bhi, Ly6Chi, Ly6Glo) from uninfected WT C57Bl/6 (blue) and fGR1 (red) were analyzed by flow cytometry for expression of A) IFNGR1 and B) the IFNγ-activated genes MHC II, CD80, and CD64. (Data were pooled from at least 3 independent experiments, 2–3 mice per group per experiment). (TIF) [file ppat.1006388.s006.tif]

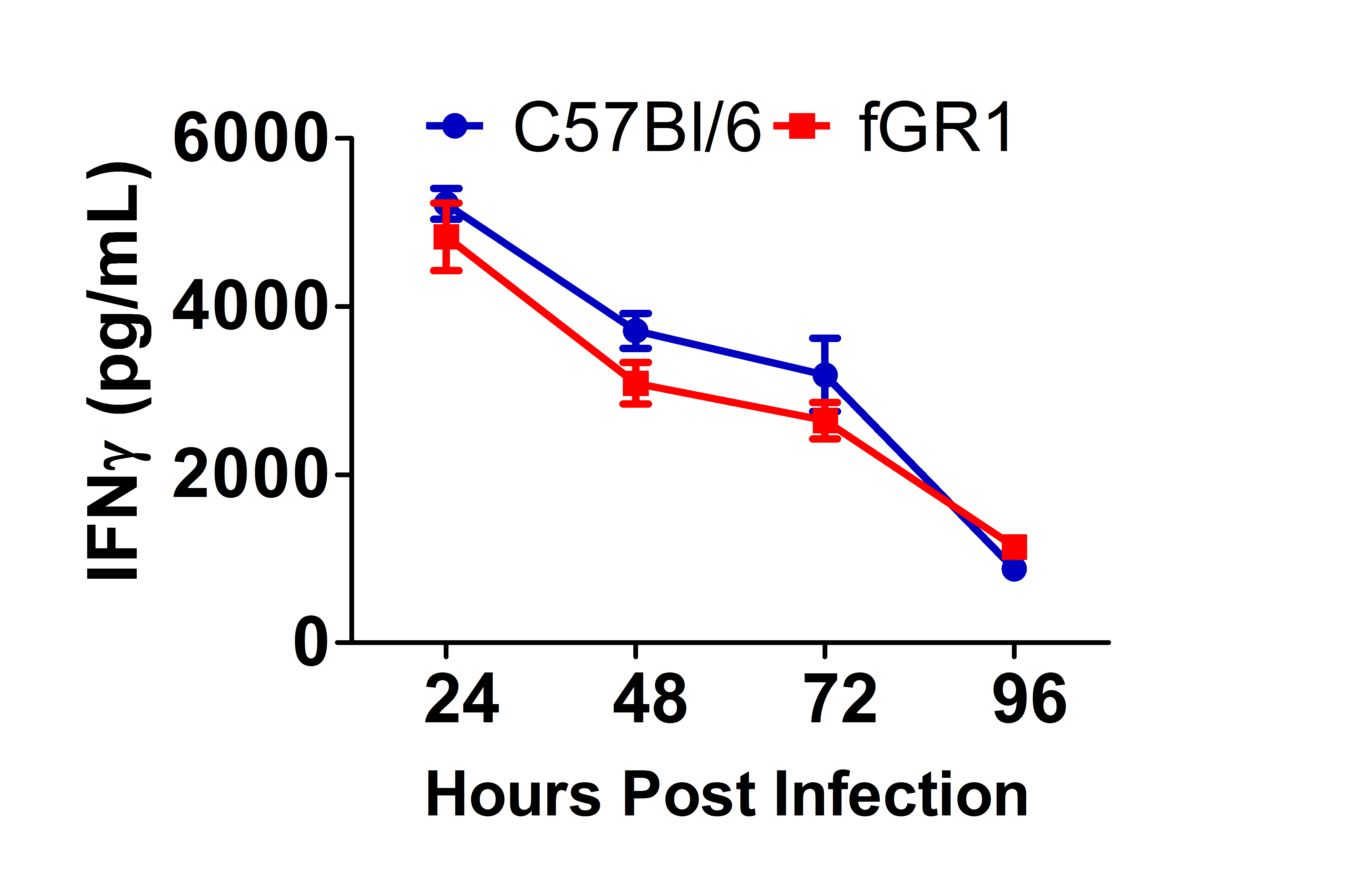

Supplement: S6 Fig — Serum was collected from WT C57Bl/6 (blue) and fGR1 (red) mice 24, 48, 72 and 96 hpi and used in an ELISA to determine the pg/mL of IFNγ. (All time points were pooled from at least 3 independent experiments, 3–5 mice per group per time point). (TIF) [file ppat.1006388.s007.tif]

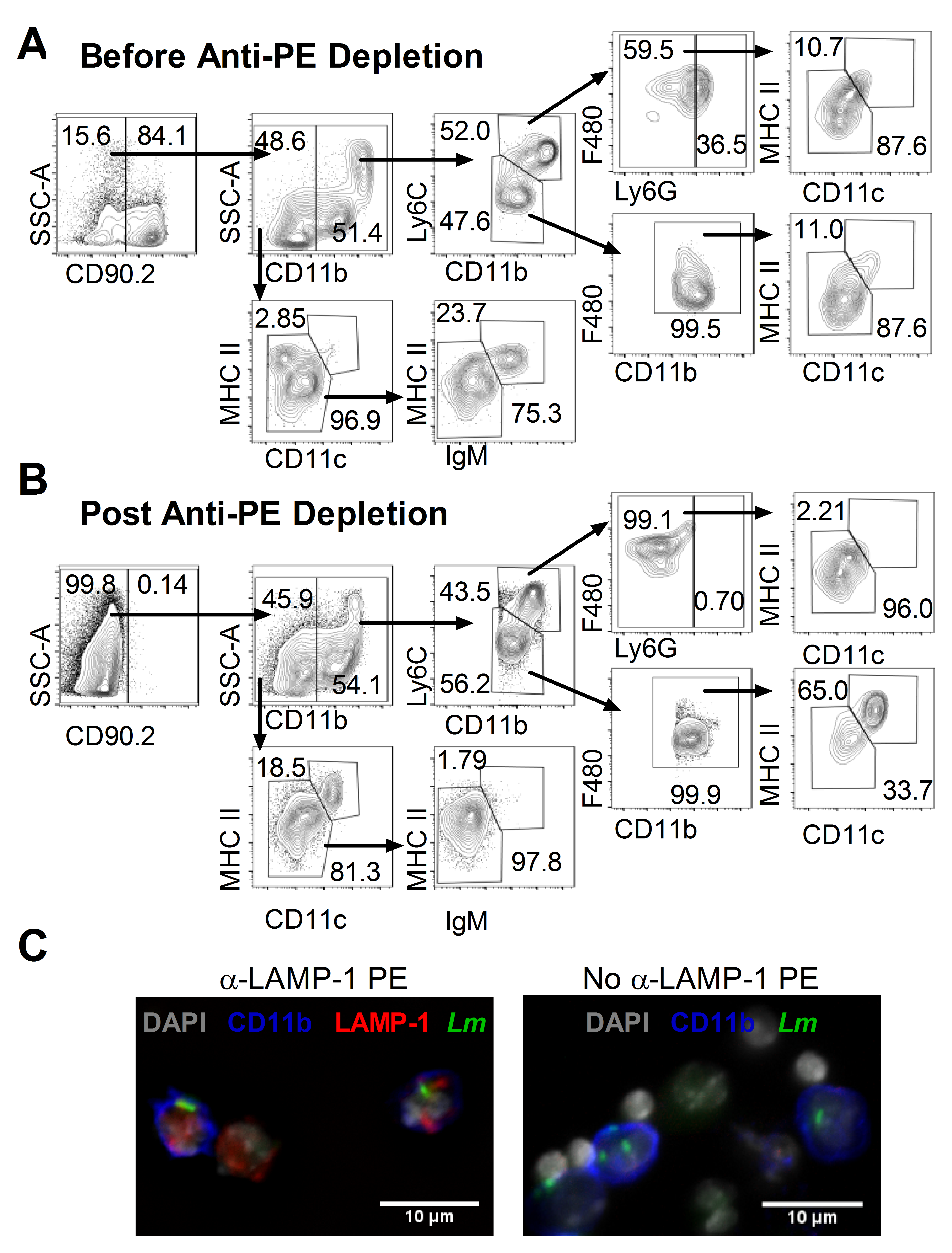

Supplement: S7 Fig — Splenocytes 60 hpi with L. monocytogenes were FACS stained with PE-congregated mAbs for CD90.2, IgM, NK1.1, and Ly6G and incubated with α-PE beads. The composition of cells was determined by flow cytometry. A) Cell populations before LS columns. B) Cells that flowed-through the columns and were collected for analyses in Fig 7. C) The purified population of cells from (B) were stained with DAPI, anti-CD11b, and anti-Lm ± anti-LAMP-1 to evaluate “carry-over” of the PE. Flow plots and images are representative of at least 3 independent experiments with 3 mice per experiment. (TIF) [file ppat.1006388.s008.tif]
